# The RNA exosome contributes to gene expression regulation during stem cell differentiation

**DOI:** 10.1093/nar/gky817

**Published:** 2018-09-13

**Authors:** Marta Lloret-Llinares, Evdoxia Karadoulama, Yun Chen, Luke A Wojenski, Geno J Villafano, Jette Bornholdt, Robin Andersson, Leighton Core, Albin Sandelin, Torben Heick Jensen

**Affiliations:** 1 Department of Molecular Biology and Genetics, Aarhus University, Denmark; 2 The Bioinformatics Centre, Department of Biology, University of Copenhagen, Denmark; 3 Biotech Research and Innovation Centre (BRIC), University of Copenhagen, Denmark; 4 Department of Molecular and Cell Biology, Institute for Systems Genomics, University of Connecticut, Storrs, CT, USA

## Abstract

Gene expression programs change during cellular transitions. It is well established that a network of transcription factors and chromatin modifiers regulate RNA levels during embryonic stem cell (ESC) differentiation, but the full impact of post-transcriptional processes remains elusive. While cytoplasmic RNA turnover mechanisms have been implicated in differentiation, the contribution of nuclear RNA decay has not been investigated. Here, we differentiate mouse ESCs, depleted for the ribonucleolytic RNA exosome, into embryoid bodies to determine to which degree RNA abundance in the two states can be attributed to changes in transcription versus RNA decay by the exosome. As a general observation, we find that exosome depletion mainly leads to the stabilization of RNAs from lowly transcribed loci, including several protein-coding genes. Depletion of the nuclear exosome cofactor RBM7 leads to similar effects. In particular, transcripts that are differentially expressed between states tend to be more exosome sensitive in the state where expression is low. We conclude that the RNA exosome contributes to down-regulation of transcripts with disparate expression, often in conjunction with transcriptional down-regulation.

## INTRODUCTION

Embryonic stem cells (ESCs) are pluripotent, self-renewing cells derived from the inner cell mass of the developing blastocyst. ESCs display a specific gene expression program, which maintains their indefinite proliferation profile while at the same time potentiating differentiation upon exposure to appropriate stimuli (1–3). Control of ESC pluripotency and differentiation potential has been extensively investigated and requires a complex network of key transcription factors (TFs) along with epigenetic mechanisms that establish the required chromatin states (2–6). Several of such studies have been based on letting ESCs differentiate spontaneously into so-called embryoid bodies (EBs), which are 3D structures that recapitulate early embryo development and express TFs that drive the cells through lineage pathways to form differentiated cell types (7–9).

While TF control of the ESC state and of gene expression regulation during differentiation is well established, the extent to which regulation occurs at the post-transcriptional level is less understood. Yet, research over recent years has attested to its relevance, revealing a role for differential mRNA processing, RNA binding proteins (RBPs) and miRNA function (6,10–13). Moreover, regulated RNA turnover may change cellular levels of transcripts with short half-lives, e.g. mRNAs encoding certain TFs (14–17) with potential relevance in dynamic systems. Indeed, estimated rates of mRNA decay in mouse ESCs (mESCs) and in human induced pluripotent stem cells (iPSCs) revealed marked changes when compared to their differentiated counterparts (18,19). In line with a central role of RNA turnover in cellular differentiation, RBPs that reduce transcript stability are involved in regulation of stem cell self-renewal and differentiation (3,10). For example, a mutagenesis screen in haploid ESCs identified Pum1, a protein that binds to 3′UTRs of mRNAs and promotes their degradation (20), as a facilitator of the exit from pluripotency via its down-regulatory impact on mRNAs encoding naive pluripotency factors (21). More recently, nonsense-mediated RNA decay (NMD) was shown to play a role during stem cell differentiation, both in mice and in humans (22,23), and finally, RNA methylation has been linked to the destabilization of mRNAs from pluripotency genes in mESCs, allowing cells to transition into their differentiated states (24,25). Although these reported examples involve RNA degradation in the cytoplasm, nuclear RNA decay also has the capacity to play an important role because of its key ability to process immature transcripts and to remove faulty and/or unstable nuclear RNAs (26–29). The latter might be particularly relevant due to the vast amount of long non-coding (lnc) RNA produced from mammalian genomes (27,30), and the impact of a number of these transcripts in key biological processes, including stem cell pluripotency and differentiation (31–33).

A central player in eukaryotic RNA turnover is the 3′-5′ exo- and endo-nucleolytic RNA exosome. Although it is present in both the nucleus and in the cytoplasm, exosome depletion appears to mostly impact nuclear RNA metabolism (34,35). Here, the exosome is involved in rRNA/snRNA/snoRNA processing, turnover of both lncRNAs and mRNAs and the selective degradation of aberrant transcripts (29,36,37). To aid in the recognition and recruitment of its multiple classes of RNA targets, the exosome associates with a number of co-factors and adaptor complexes; e.g. two nucleoplasmic decay pathways guided by the nuclear exosome targeting (NEXT) complex and the poly(A) tail exosome targeting (PAXT) connection direct the exosomal decay of shorter immature RNAs and longer polyadenylated transcript species, respectively (38,39). Of particular interest for this study, the exosome may regulate levels of specific transcripts in response to environmental cues or during developmental programs (29). Two such examples include: i) meiotic mRNAs in *Schizosaccharomyces pombe* cells, which are subject to exosomal decay during mitotic growth in a process mediated by the Mmi1 protein (40,41) and ii) the contribution of the human exosome to the maintenance of epidermal progenitor cells through its selective degradation of mRNAs encoding TFs necessary for differentiation (42).

In this study, we employ high throughput transcription and RNA abundance measurements to investigate how the RNA exosome contributes to the shaping of transcriptomes during the differentiation of ESCs into EBs. Our results demonstrate a marked role of the exosome in controlling expression levels, especially of transcripts deriving from lowly transcribed loci, including protein-coding genes. Such exosome function also contributes to dampen RNA output from downregulated genes during the differentiation process.

## MATERIALS AND METHODS

### ESC culture and differentiation

E14 ESCs were grown on 0.2% gelatin coated plates in 2i medium (43,44) containing: DMEM/F12 and Neurobasal 1:1, N2 supplement, B27 supplement, 2 mM glutaMax, penicilin/streptomycin, 1 mM sodyum pyruvate, 50 nM 2-mercaptoethanol, nonessential amino acids (all of the above from Gibco), LIF, 3 μM GSK3 inhibitor (CHIR-99021) and 1 μM MEK inhibitor (PD0325901). For differentiation into embryoid bodies, the medium was the same, except for the LIF, GSK3 inhibitor and MEK inhibitor and it contained 10% FBS (Gibco). 20 μl drops containing ∼1000 cells each were hung on lids of tissue culture plates for 2 days and subsequently transferred to a Petri dish for an additional 24 h.

### Depletion experiments

Knockdowns were performed using stably integrated shRNAs. E14 cells were transduced with the following pLKO vectors (from sigma): SHC002 (scrambled control), NM_025513.1-909s1c1 (RRP40), NM_144948.2-1021s1c1 (RBM7). Cells were transduced with lentiviral particles for 16 h and selected with 2 μg/μl puromycin 48 h after transduction.

### Western blotting analysis

Cells were washed twice in PBS, harvested and centrifuged for 5 min at 1500 rpm. Then they were lysed in RSB100, containing 0.5% Triton X-100 (10 mM Tris pH 7.4, 100 mM NaCl, 2.5 mM MgCl_2_, 0.5% Triton X-100). SDS-PAGE and western blotting analysis were carried out according to standard procedures using the following antibodies: anti-RRP40 (Proteintech, 15062-1-AP) at 1:4000, anti-RBM7 (Sigma, protein atlas, HPA013993) at 1:500, anti-alpha-tubulin (Rockland, 600-401-880) at 1:5000. Secondary HRP goat anti-rabbit (Dako, P0448) antibody was used at 1:5000.

### RNA isolation

RNA was isolated using TRIzol (Ambion) and treated with TURBO DNAse (Ambion) according to the manufacturer's instructions.

### RT-qPCR analysis

cDNA was prepared with the SuperScript II kit (Invitrogen), using 1 μM oligo dT18 and 5 ng/μl random hexamers. To evaluate genomic DNA contamination, a negative control was prepared in parallel by treating the same amount of RNA in the same way but without adding the reverse transcriptase enzyme. qPCR was performed with Platinum SYBR Green qPCR SuperMix-UDG (Invitrogen) in a MX3000P (Agilent technologies) machine. The primer sequences used for qPCR are listed in Supplementary Table S2.

### RNAseq library preparation and data processing

100 pg each of in-house made spike-in RNAs (Supplementary Table S3) were added to 5 μg of total RNA pre-treated with the Ribo-Zero magnetic kit to deplete rRNA. Libraries were constructed with the TruSeq Stranded mRNA LT Sample Prep Kit (Illumina) and sequenced on an Illumina HiSeqTM4000 system. Quality control of sequence reads was done using FastQC v0.11.2 (http://www.bioinformatics.babraham.ac.uk/projects/fastqc/). Illumina adaptors, low quality bases, the first 12 bases and reads smaller than 25 nt were removed with Trimmomatic v0.32, using settings ‘ILLUMINACLIP:<TrueSeq3_PE_2>:2:30:10 HEADCROP:12 LEADING:22 SLIDINGWINDOW:4:22 MINLEN:25’ (45). Reads were mapped using HISAT2 v2.0.4 (46), against the mouse reference genome (mm10) and in-house spike-in sequences. For read mapping, we provided HISAT with a list of GENCODE (47)-annotated splice sites, setting the maximum fragment length to 1000 and using the –rf parameter for the upstream/downstream mate orientation, and using default settings for the rest of the parameters. Uniquely mapping and properly paired reads were selected using Samtools v0.1.17 (48) and used for downstream analysis. genomecov from Bedtools v2.23.0 (49) was used to calculate strand specific per base genome coverage in bedgraph format. Bedgraph files were converted into bigwig format using the UCSC Genome Browser Utility ‘bedGraphToBigWig’ (50).

### PROseq library preparation and data processing

PROseq libraries from ∼1 million permeabilized cells were constructed as in (51), with the following modifications. After the 3′-ligation, the cap-removal, end repair and 5′-ligation reactions were all performed on the beads by doubling of reaction volumes. After the 5′-ligation the beads were washed, and samples eluted and reverse transcribed. Test amplifications of serial dilutions from each RT reaction were used to determine the number of cycles for full amplification, with a maximum of 15 cycles. Fully amplified libraries were PAGE purified on a 8% PAGE gel, quantified and sized by Qbit and Agilent tapestation, respectively and sequenced on an Illumina NextSeq 500 (75 bp—high throughput kit) at the Center for Genome Innovation (Storrs, CT, USA). Adapter sequences were trimmed with FASTX-Toolkit and filtered for a minimum of 15 bases. All reads were then trimmed to a maximum of 36 bases and reverse complimented. Reads were first mapped with Bowtie (52) to a copy of the mouse rDNA repeat (GenBank: BK000964.1) with the -K1 option, and unaligned reads were then mapped to the mouse genome (mm10) filtering for unique matches. Only the final 3′-base representing the position of polymerase was reported to output files used in all analyses.

### CAGE library preparation and data processing

RNA from each of the biological duplicates were quality controlled using a Bioanalyzer. RIN scores were between 9.7 and 10. CAGE libraries were prepared using the protocol by (53) with an input of 3 μg of total RNA. Samples were run individually, but prior to sequencing, four CAGE libraries with different barcodes were pooled and applied to the same sequencing lane. Sequencing of the libraries was performed on a HiSeq2000 instrument from Illumina at the National High-Throughput DNA Sequencing Centre, University of Copenhagen. To compensate for the low complexity of 5′ends in the CAGE libraries, 30% Phi-X spike-ins were added to each sequencing lane, as recommended by Illumina. CAGE reads were assigned to their respective originating sample according to identically matching barcodes. Using the FASTX Toolkit (v0.0.13), assigned reads were i) 5′-end trimmed to remove linker sequences (9+2 bp to account for the CAGE protocol G-bias), ii) 3′-end trimmed to a length of 25 bp and iii) filtered for a minimum sequencing quality of 30 in 50% of the bases. Trimmed reads were mapped using Bowtie (52) (Version 0.12.7) with parameters –t –best –strata –v –k 10 –y –p 6 –phred33-quals –chunksmbs 512 –e 120 –q –un to ASM294v2.26. To obtain bp resolution CAGE TSSs (CTSSs), the number of CAGE tag 5′ends were counted for each genomic position. Tag clusters, used for differential expression, were constructed by merging nearby 5′ends on the same strand as in (54). Expression levels of tag clusters were normalized to tags per million mapped reads (TPM).

### Gene annotation

The mouse GENCODE annotation version M9 (47) was used to annotate RNAs by biotypes, used for downstream analysis. In addition, sets of PROMPTs and eRNAs were defined to expand the set of exosome sensitive RNAs. A total of 8453 PROMPTs were defined using 24561 primary and alternative TSSs derived from the CAGE data, guiding the definition of PROMPT TSSs. Starting from CAGE tag clusters falling within 100bp of GENCODE genic TSSs on the gene strand, PROMPTs were defined as the closest upstream CAGE tag clusters to the above TSSs on the opposite strand, up to 2 kb distant of the genic TSS. For measuring the expression of PROMPTs, we counted RNAseq tags in the first 2 kb region downstream of the PROMPT TSS on the PROMPT strand. To define enhancers and eRNA locations, we used already established enhancer regions, and eRNA TSSs from FANTOM 5 mouse data (55). We quantified their expression in the 1kb region downstream of eRNA TSSs, on the same strand (two regions per enhancer, because of their bidirectional TSSs). The transcription of PROMPTs and eRNAs was measured in the same way, but using PROseq data.

### RNAseq quantification and normalization

RNA expression in control, RRP40- and RBM7-depleted libraries was quantified by counting uniquely mapped and properly paired RNAseq fragments that overlapped exons on the relevant strand and summarized on the gene level using the Rsubread package (56). Fragment counts were converted to normalized expression values using the median ratio normalization of the DESeq2 R package (57). The size factors used for the normalization were calculated using spike-ins counts. This quantification was done for all genes in the mouse GENCODE annotation version M9 (47) and for the PROMPT and eRNA sets defined as above. RNAs with normalized expression values above 0 in all three replicates of at least one experimental condition were quantile normalized across all samples (58), assuming that gene expression follows the same distribution in both mESCs and EBd3s. We used the mean gene expression of control mESCs replicates as a reference for normalizing both the mESC and EBd3 data sets, including the factor depleted (RRP40, RBM7) libraries.

### PROseq quantification and normalization

The PROseq signals in control and factor depleted (RRP40, RBM7) libraries were quantified by counting PROseq fragments that overlapped genes (specifically, from the most 5′TSS to the most distal 3′end of the gene model, including exons and introns) on the relevant strand using the Rsubread package (56). Fragment counts were converted to normalized expression values using the median ratio normalization method in the DESeq2 R package (57). This quantification was done for all genes in the mouse GENCODE annotation M9 (47) and for the PROMPT and eRNA sets, as for RNAseq data as described above. Only genes with normalized expression values >0 in both replicates of at least one condition were used in the analysis. The normalized expression values were corrected for a batch effect due to different sequencing times using the ComBat function from the sva R package (https://doi.org/doi:10.18129/B9.bioc.sva). The design matrix used in the batch correction model included cell type and knockdown type and the sequencing time as factors. The batch effect corrected normalized expression values were quantile normalized as described above for RNA-seq data, and the first (lowest) percentile of the data was excluded.

### CAGE quantification and normalization

CAGE tag clusters with TPM>0 in both replicates were selected and the TPM expression values were quantile normalized as described above for RNAseq data. To avoid analyzing lowly expressed TSSs within genes, we removed tag clusters falling within genes on the same strand that contributed <1.5% of the total CAGE expression, using the average of replicates.

### Sensitivity definition

Based on the strand-specific expression described above, the RRP40 and RBM7 sensitivity of an RNA was defined as follows:

(1) \begin{eqnarray*} {\rm sensitivit}{{\rm y}_{{\rm KD}}}= \frac{{{\rm expressio}{{\rm n}_{{\rm KD}\ {\rm library}}} - \ {\rm expressio}{{\rm n}_{{\rm CTRL}\ {\rm library}}}}}{{{\rm expressio}{{\rm n}_{{\rm KD}\ {\rm library}}}}} \end{eqnarray*}

where expression values are either normalized RNAseq or CAGE signal, and all negative values were set to 0.

### Statistics and visualizations

Visualizations were made using mainly the ggplot2 R package (59). Statistical tests were done in the environment of the R Project for Statistical Computing (https://www.r-project.org). Genome browser plots were made using the UCSC browser (60).

## RESULTS

### Exosome depletion in ESCs and EBs

**Figure 1.**
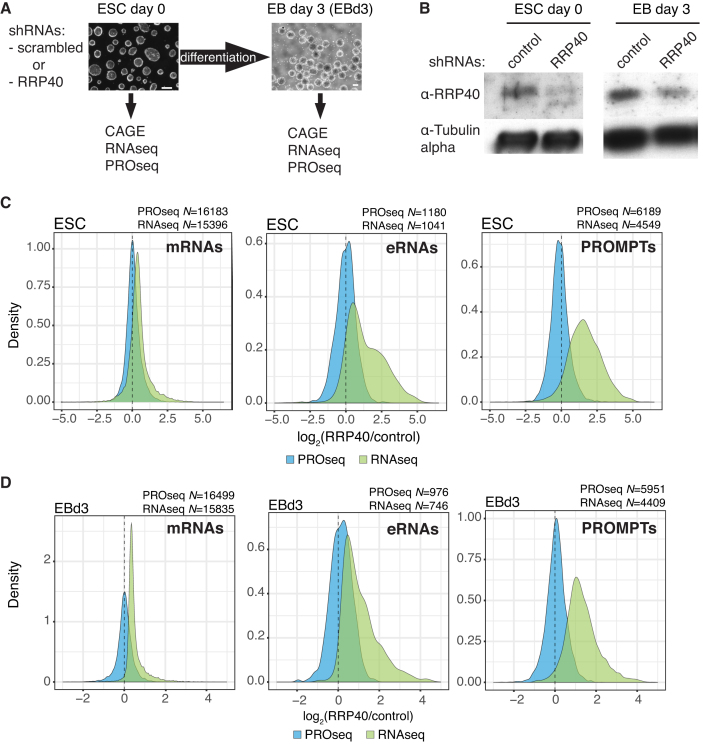
Effects of RRP40 depletion in ESCs and EBs. (**A**) Schematic representation of the experimental procedure and data collection. Scrambled control or RRP40-specific shRNAs were introduced using lentiviral vectors into ESCs, after which cells were differentiated into EB for 3 days. From each cell state and shRNA condition, CAGE, RNAseq and PROseq data were collected. The scale bar on the lower right corner of the images represents 100 μm (ESC) and 200 μm (EBd3). (**B**) Western blotting analysis of RRP40 levels in ESCs treated with the indicated shRNAs and differentiated for 0 (left) or 3 (right) days. Tubulin was used as a loading control. (**C**) Distributions (y-axis) of log_2_ fold changes (x-axis) between RNAseq (green) or PROseq (blue) values from RRP40-depleted versus control ESC samples. The RNA types measured and their numbers are indicated above each panel. The average of all replicates is shown. (**D**) As in (C) but for the EBd3 samples.

To examine the contribution of the RNA exosome to the early differentiation of ESCs, we used short hairpin (sh) RNAs to deplete its core subunit RRP40 followed by differentiation of the ESCs into EBs for 3 days (EBd3, Figure 1A and B). Cells depleted for RRP40 formed EBs of a similar size and morphology as the control cells expressing a scrambled shRNA (Supplementary Figure S1A). Moreover, the expression of several pluripotency and differentiation markers was largely unaffected by the decreased RRP40 levels (Supplementary Figure S1B). Notably, the RRP40 depletion efficiency was lower in EBs than in ESCs (Figure 1B and Supplementary Figure S1C), which is probably due to selective pressure against cells with low levels of RRP40.

From both ESC and EBd3 differentiation stages, total RNA was harvested, depleted of rRNA, and subjected to regular RNA sequencing (RNAseq) and Cap Analysis of Gene Expression (CAGE) sequencing to capture capped RNA 5′ends (53) (Figure 1A). Moreover, transcription activity in the same cell stages was measured directly by Precision nuclear Run-On sequencing (PROseq) (51,61). RNAseq (triplicate), CAGE (duplicate) and PROseq (duplicate) samples all displayed good replicate agreements (Supplementary Figure S1D). To correct for the reduced RRP40 depletion efficiency in the EBd3 state (Figure 1B and Supplementary Figure S1C), read distributions from each applied sequencing method were quantile-normalized using the ESC data as reference distributions (see Materials and Methods). For downstream analysis, we only considered RNAs/genes captured by all of the replicates in at least one condition (ESC or EBd3) in each type of sequencing experiment. Moreover, in order to measure the transcription activity from, or RNA abundance of, a given gene, all GENCODE transcript models for the relevant transcription unit were merged. For simplicity, we refer to these merged transcript models as ‘genes’ and their products as ‘RNAs’ in the rest of the text. Finally, to complement the RT-qPCR data from Supplementary Figure S1B, RNAseq data were used to interrogate a larger set of mRNAs expressed in the ESC and EBd3 states (62,63), which did not reveal any major differences between shRNA-RRP40 and shRNA-control cells (Supplementary Figure S1E and Table S1). Thus, analyzed cells were pluripotent and formed EBs with similar characteristics.

Depletion of the exosome is predicted to stabilize a set of RNAs without affecting their transcription levels. Consistently, RRP40 depletion resulted in elevated RNA levels of known exosome targets, such as enhancer RNAs (eRNAs) and PROMoter uPstream Transcripts (PROMPTs) (64–67), without any overall effect on transcription, as judged by PROseq signal, at these loci (Figure 1C and D). We therefore conclude that lowered exosome activity impacts RNA levels globally with no global effect on transcription. Hence, comparison of RNAseq and PROseq data allowed for the discrimination between changes in RNA levels instigated by altered transcription or RNA turnover during mESC differentiation.

### Lowly expressed RNAs are preferential exosome targets

**Figure 2.**
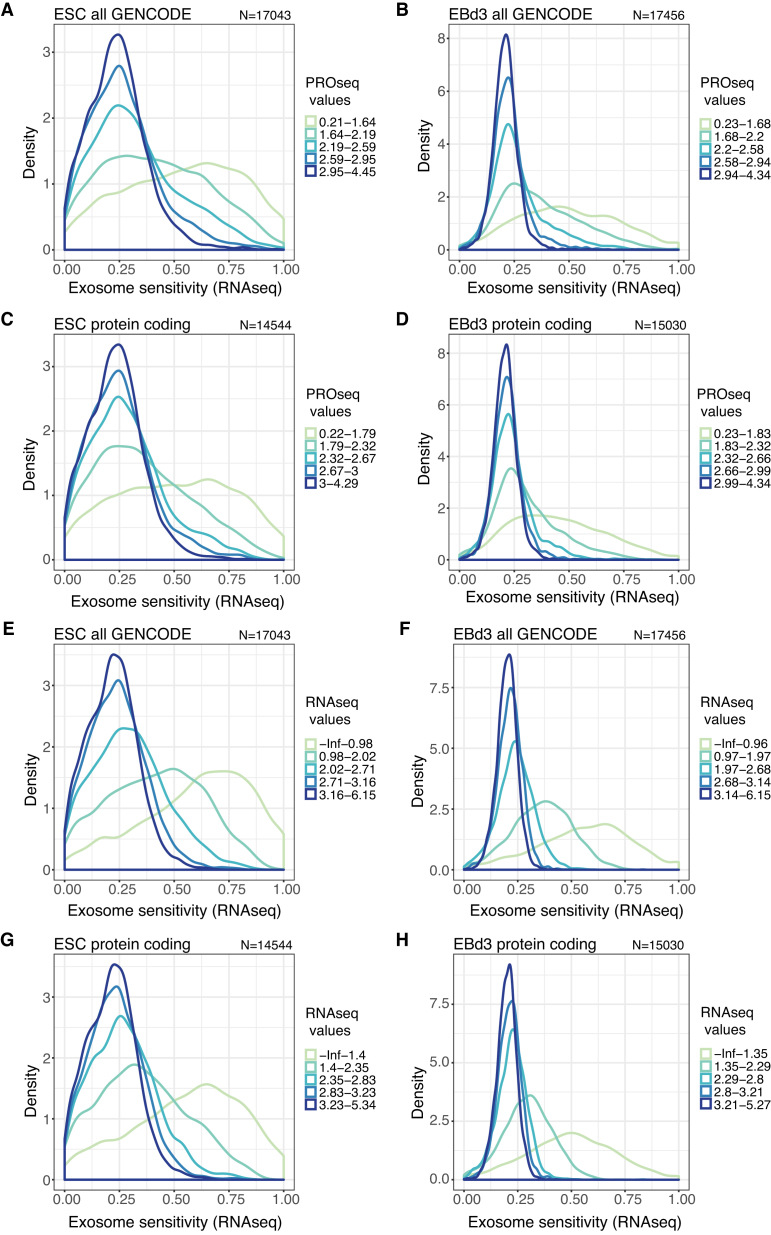
Transcripts from lowly expressed genes are preferentially exosome sensitive. (**A** and **B**) Distributions of RNA exosome sensitivities calculated from RNAseq data (see Methods) for RNAs from all GENCODE annotated genes, quintile-stratified based on their normalized PROseq values in control ESC (**A**) or EBd3 (**B**) cells. (**C** and **D**) As in (A and B) but for RNAs from GENCODE-annotated protein-coding genes only. (**E**–**H**) Equivalent to panels (A–D) but with quintile-stratification based on normalized RNAseq values in the respective control samples. For all plots, the number of genes/RNAs (*N*) included is indicated above each panel. Quintiles are defined by the thresholds shown in the legends to the right of plots.

Before comparing the two examined cell states, we investigated if there was a general relation between transcription level and exosome sensitivity. To do this, we divided all GENCODE annotated genes in quintiles according to their normalized PROseq read counts from control cells and calculated the exosome sensitivity of the respective RNAs from the RNAseq data on a scale from 0 to 1, where 0 indicates no expression difference between control and exosome-depletion samples and 1 indicates exclusive expression upon exosome depletion (see Materials and Methods). For both ESC and EBd3 states, RNAs produced by genes with higher transcription levels exhibited lower exosome sensitivity (Figure 2A and B). Conversely, exosome sensitivity increased for RNAs derived from genes with lower transcription levels.

We initially considered two different technical explanations for this strong correlation. Firstly, as the GENCODE annotation is wide-spanning and covers numerous ncRNAs, the observed tendency could be driven by lowly expressed and exosome-sensitive lncRNAs. However, the trend persisted when only considering RNAs deriving from protein coding genes (Figure 2C and D). Secondly, the used sensitivity statistic forces values into a narrow scale from 0 to 1 (see Materials and Methods). To control that this did not lead to skewing of the data, we calculated the distribution of log_2_ fold changes of RNAseq signals between the RRP40-depleted and control samples. This yielded a similar trend; RNAs expressed from all lowly transcribed GENCODE-annotated genes were more exosome sensitive (Supplementary Figure S2A and S2B), which was also the case when only assessing RNA from protein-coding genes (Supplementary Figure S2C and S2D).

Although RNA and transcription levels are generally positively correlated, deviations may occur due to post-transcriptional events. We therefore asked whether the relationship between low transcription activity and high exosome sensitivity would also be observable when using RNAseq levels to stratify genes into quintiles. For both investigated cell states and for both all GENCODE genes (Figure 2E and F, Supplementary Figure S2E and S2F) and only protein coding genes (Figure 2G and H, Supplementary Figure S2G and S2H), this appeared to be the case. Exosome depletion mostly affects nuclear RNAs (34,35). To confirm this, we depleted the nuclear exosome cofactor and NEXT component RBM7 (Supplementary Figure S2I) (38) in ESCs and differentiated these into EBs for 3 days, sampling material for RNAseq and PROseq analyses as above. In line with our RRP40-depletion results, transcripts deriving from lowly transcribed genes (Supplementary Figure S2J, S2K, S2L and S2M), and from genes yielding low levels of RNA (Supplementary Figure S2N, S2O, S2P and S2Q), exhibited higher RBM7 sensitivity. This indicates that these RNAs are targeted by nuclear decay, at least in part, via the NEXT complex.

We conclude that lowly expressed genes, as measured by either transcription or RNA data, produce transcripts that are more exosome sensitive than those expressed from more active units. Although the mechanism underlying this relationship is not clear (see Discussion), it raises the interesting possibility that the RNA exosome might contribute to the removal of transcripts from already transcriptionally down-regulated genes.

### Differentially expressed RNAs are subject to exosome decay in the cell state where they are lowly expressed

**Figure 3.**
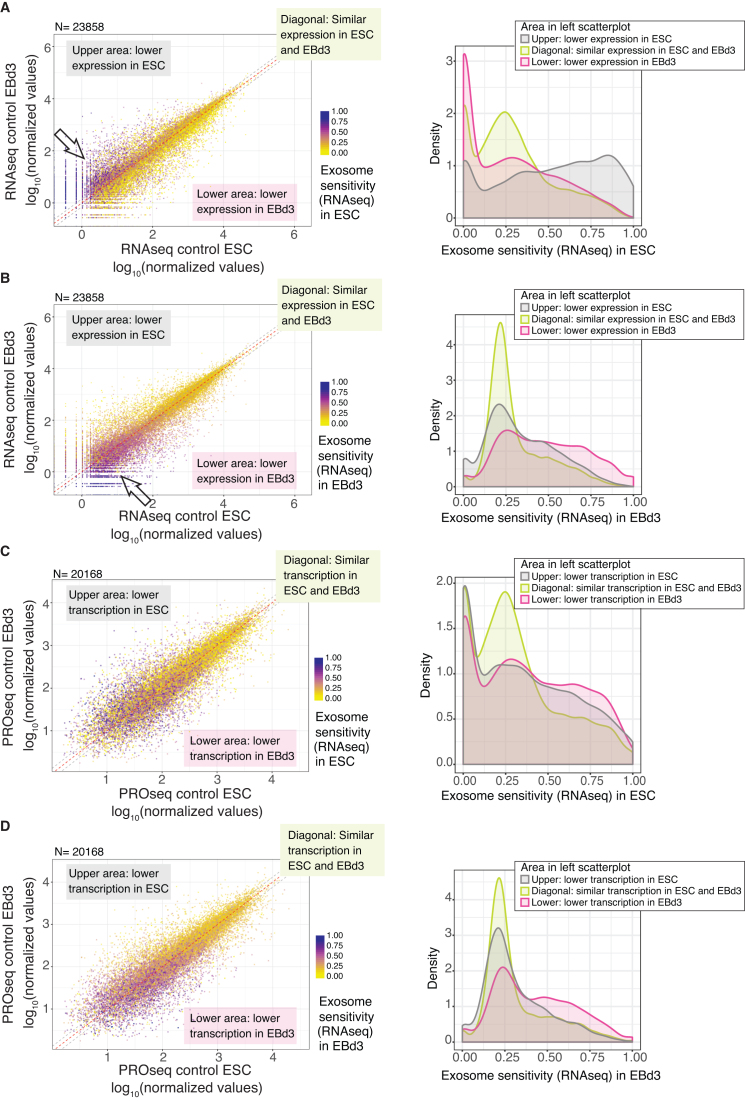
Transcripts tend to be exosome sensitive in the cell state where they are most lowly expressed. (**A**) Left panel: log_10_ normalized RNAseq values for RNAs from all GENCODE-annotated genes in control EBd3 (y-axis) and control ESC (x-axis) states, coloured according to their exosome sensitivity in the ESC state as calculated from RNAseq data (as shown on the legend to the right: purple denotes the most exosome-sensitive RNAs). The white arrow emphasizes particularly exosome-sensitive RNAs. The red dashed line marks equal expression levels between EBd3 and ESCs. Based on the red dashed lines, three regions of the plot were defined, indicated by callouts and grey dotted lines, which are further analyzed in the right panel: upper area (region above upper grey line, grey line in the right panel) contains transcripts with lower expression in ESCs; lower area (region below lower grey line, pink line in the right panel) contains transcripts with lower expression in EBd3; diagonal (region between grey lines, green line in the right panel) contains transcripts with similar expression in ESC and EBd3. Right panel: Densities of RRP40 sensitivity in ESCs for transcripts falling into the three areas defined in the left panel. (**B**) As in A, but with exosome sensitivity calculated from the EBd3 state. (**C**) As in A, but signals on x- and y-axes in the left panel are based on PROseq data. (**D**) As in C, but with exosome sensitivity calculated from the EBd3 state. For all plots, the number of genes/RNAs (*N*) included is indicated above each panel.

We next wondered how the relationship between low transcript expression level and high exosome sensitivity would relate to the differentiation process. To address this, we plotted RNAseq-derived expression levels in the ESC versus EBd3 states in control cells and indicated by color-coding the exosome-sensitivity of the RNAs in either the ESC (Figure 3A, left panel) or EBd3 (Figure 3B, left panel) samples. Interestingly, the most sensitive RNAs in ESCs were not only lowly expressed, but also less expressed in the ESC than in the EBd3 state (Figure 3A, left panel; note arrow and purple dots above the diagonal, also density plotted as the grey area in the right figure panel). Conversely, the most sensitive RNAs in the EBd3 state were more lowly expressed in EBd3 cells than in ESCs (Figure 3B, left panel; note arrow and purple dots below the diagonal, also density plotted as the pink area in the right figure panel). This effect was less pronounced in the EBd3 state, likely caused by its less efficient RRP40 depletion and the employed quantile-normalization approach. Importantly, RNAs whose levels were higher in the interrogated cell state were not as exosome sensitive in that state, even if their expression levels were low (Figure 3A, left panel; dots below the diagonal, also density plotted as the pink area in right figure panel; and Figure 3B; left panel; dots above the diagonal, also density plotted as the grey area in right figure panel). Plotting of the CAGE data showed a similar relationship of higher exosome sensitivity of RNAs in the cell state where they were lower expressed (Supplementary Figure S3A and S3B). RBM7 depletion resulted in analogous effects (Supplementary Figure S3C, S3D, S3E and S3F), suggesting that the relevant RNAs are degraded in the nucleus. It therefore appears that the RNA exosome contributes to minimizing RNA levels from genes that are already specifically downregulated in one cell state.

We next analyzed transcription levels (obtained from PROseq data) in the two cell states and related it to RNA exosome sensitivity (obtained from RNAseq data) as above. Consistent with our previous finding (Figure 2), RNAs deriving from genes with lower transcription levels tended to be more exosome sensitive. However, there was no clear tendency for RNAs that were less transcribed in one cell state to also be more exosome sensitive in the same condition (Figure 3C and D). Similar results were obtained from RBM7 depleted cells (Supplementary Figure S3G and S3H). Taken together, our analyses therefore imply that exosomal turnover contributes to the specific depletion of transcripts from genes needing a lower RNA output in one of the differentiation states. This appears to be independent on whether transcription levels (as opposed to RNA levels) change between states. These results indicate that there might be a specific targeting to the exosome for downregulated transcripts that cannot solely be explained by the observed correlation between low expression/transcription levels and exosome sensitivity.

### Relative contributions of transcription and RNA degradation during differentiation

**Figure 4.**
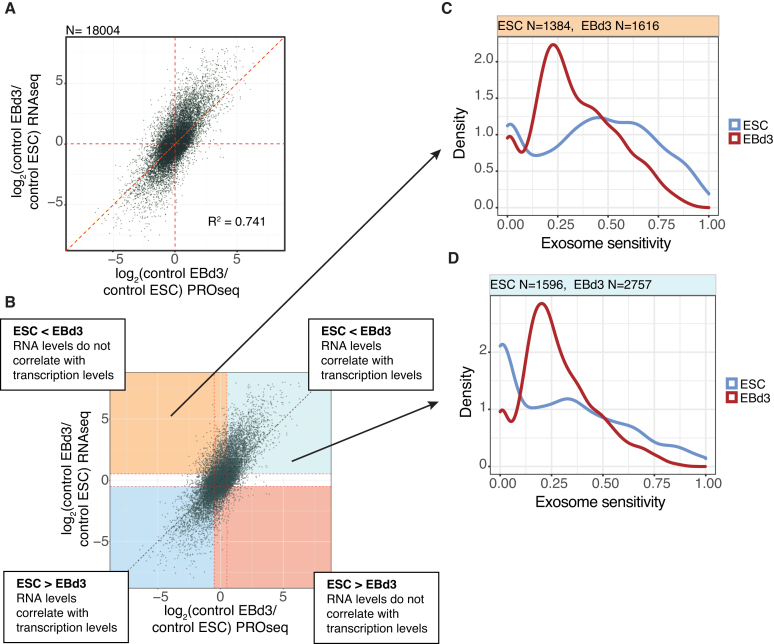
Contribution of transcription and exosome decay to RNA changes. (**A**) Scatter plot of log_2_ fold changes between RNAseq values of all GENCODE annotated genes in EBd3 versus ESC samples (y-axis) against log_2_ fold changes between PROseq values in EBd3 versus ESC samples (x-axis). Pearson's R square value is indicated. Red dashed lines indicate *x* = 0, *y* = 0 and *y* = *x*. (**B**) Same plot as in (A) but defining subsets of the data based on up- and down-regulation between EBd3 versus ESC, and whether RNAseq and PROseq value changes correlate positively (blues) or not (red-orange), as indicated by callouts. (**C**) Density plots of RNAseq-calculated exosome sensitivities from ESC (blue line) and EBd3 (red line), analyzing transcripts downregulated in ESC that have no correlation between RNAseq and PROseq changes (top-left quadrant from (B). (**D**) Density plots as in (C) but analyzing transcripts downregulated in ESC that have correlating RNAseq and PROseq changes (top-right quadrant from (B)). The number of genes/RNAs (*N*) included is indicated above each panel.

Data presented so far imply that the RNA exosome contributes to controlling RNA levels during cellular differentiation. To more directly compare the contributions of transcription versus RNA degradation, we calculated the log_2_ fold changes between the EBd3 and ESC states for all genes using RNAseq and PROseq data plotted against each other (Figure 4A). RNAseq and PROseq signal fold changes were well correlated (*R*^2^ = 0.74), indicating that most changes in RNA levels can be explained by altered transcription. However, a subset of genes with unchanged transcription levels between states (Figure 4A, dots positioned around 0 on the x-axis, but spread along the y-axis) differed in their transcript levels, implying post-transcriptional regulation.

Due to the higher exosome sensitivity towards RNAs in the cell state with the lowest transcript levels (Figure 3), we stratified genes according to whether they were upregulated (log_2_(ctrl EBd3/ctrl ESC) > 0.5, upper part of Figure 4B) or downregulated (log_2_(ctrl EBd3/ctrl ESC) < −0.5, lower part of Figure 4B) during differentiation, as measured by RNAseq. We further divided genes according to whether changes in transcription (PROseq data) and RNA levels (RNAseq data) correlated positively (Figure 4B, blue areas) or not (Figure 4B, orange and red areas). When RNA and transcription changes do not correlate, RNA turnover might predominate in controlling final RNA levels. To assess this, we analyzed the exosome sensitivity of transcripts in each cell state for the four established gene subsets (Figure 4B), but only considered genes that were not transcriptionally upregulated in the RRP40-depleted cells (log_2_(RRP40/ctrl) < 0.5 in PROseq data) to avoid analyzing indirect effects. Genes producing RNAs that were upregulated during differentiation (less RNAseq signals in ESC than in EBd3 samples), but for which the increase in RNA levels could not be explained by increased transcription (Figure 4B, top left quadrant), showed considerably higher exosome sensitivity in the ESC than in the EBd3 state (Figure 4C). In contrast, RNAs whose changes could be explained by altered transcription (Figure 4B, top right quadrant) were generally less exosome sensitive (Figure 4D, compare ESC sensitivity (blue line) to 4C). However, there were also transcripts with high exosome sensitivity (>0.5 in Figure 4D), for which transcription and post-transcription changes appeared to act together in downregulating gene expression. For genes whose RNAs were downregulated during differentiation, the effects were more subtle (again possibly due to the diminished RRP40 depletion in EBd3 cells). However, for both groups, exosome sensitivities were higher in the EBd3 state (Supplementary Figure S4A and S4B, compare red and blue lines), in which the RNAs showed lower expression levels. We conclude that exosomal turnover of RNA plays a role during the differentiation process and that it can act both independent of, and in concert with, transcription changes.

### Defining the targets of RNA turnover during ESC differentiation

**Figure 5.**
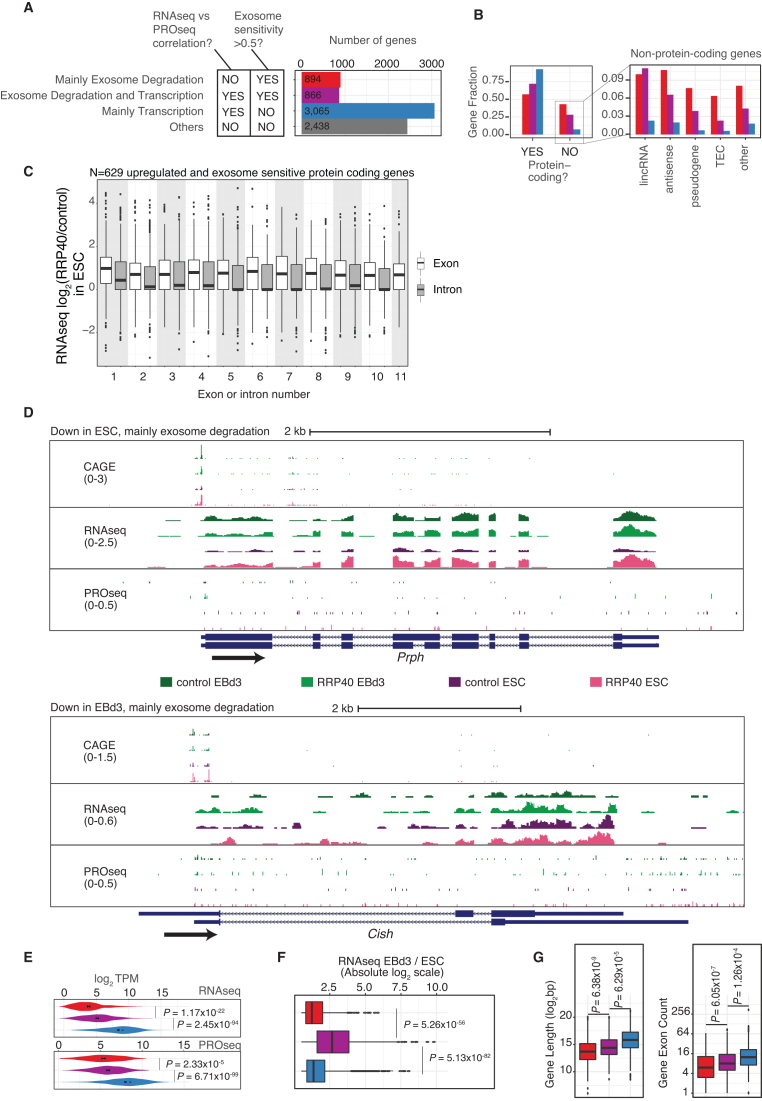
Defining the nature of RNA exosome targets. (**A**) Definition of gene/RNA classes. The left schematic shows the set of rules used for defining whether changes in RNA levels are mostly driven by transcription, exosome degradation or both. Only transcripts with an absolute log_2_ fold change of > 0.5 between ESCs and EBd3 were analyzed. Bar plots to the right show the numbers of genes/RNAs in each class. Note that bar colors were used consistently in Figure 5 to indicate the classes, only the ‘other’ class was not further analyzed. (**B**) Left panel: fractions of coding and non-coding genes/RNAs from the categories in (A). Right-panel: fractions of different types of non-coding genes/RNAs from the left panel. (**C**) RNAseq log_2_ fold changes between RRP40 depleted and control ESC samples along each exon (white) and intron (gray) of pre-mRNAs from the classes ‘mainly exosome degradation’ and ‘combination transcription and exosome degradation’ from (A). The number of transcripts analyzed (N) is indicated above the panel. (**D**) UCSC genome browser (60) examples of genes whose transcripts are downregulated in ESC (top, *Prph* locus) or EBd3 (bottom, *Cish* locus) samples, mainly due to exosome degradation. Genome browser tracks show, from top to bottom, signal intensities of CAGE, RNAseq and PROseq samples for each experimental condition on the relevant strand (as indicated by color). Numbers to the left indicate the scale for the respective data type. Bottom track shows GENCODE annotation. Arrow indicates the direction of transcription. The CAGE tracks show pooled data from the two replicates and the RNAseq and PROseq tracks show one of the replicates. (**E**) Distribution of RNAseq (top) and PROseq (bottom) average signals (normalized log_2_(TPM)) for RNAs in the classes from panel (A). *P*-values indicate results from Mann–Whitney two-sided tests between distributions. (**F**) Distributions of absolute log_2_(EBd3 versus ESC fold change) for RNAs in the classes from panel (A). *P*-values indicate results from Mann–Whitney two-sided tests between distributions. (**G**) Left panel: Distributions of gene lengths (log_2_(bp), including introns) (left). Right panel: exon count distributions for transcripts in the classes from panel (A), log_2_ scaled. *P*-values indicate results from Mann–Whitney two-sided tests between distributions.

To further characterize how RNA turnover might complement transcription regulation during ESC differentiation, we defined three gene classes based on Figure 4B as follows: (i) genes whose expression was mainly regulated by exosome degradation (transcript with exosome sensitivity in the cell state where they were downregulated >0.5 and whose genes showed no correlation between RNAseq and PROseq signal changes; red fills in Figure 5A and examples in Supplementary Figure S5A left panels), (ii) genes whose expression was regulated by a combination of exosome degradation and transcription downregulation (transcript exosome sensitivity in the cell state where they were downregulated >0.5 and a clear correlation between RNAseq and PROseq signal changes; purple fills in Figure 5A and examples in Supplementary Figure S5A right panels) and (iii) genes whose expression was mainly regulated by transcription (transcript exosome sensitivity in the cell state where they were downregulated <0.5 and a clear correlation between RNAseq and PROseq signal changes; blue fills in Figure 5A). Some of these exosome sensitive RNAs were validated by RT-qPCR (Supplementary Figure S5B). Genes that did not fall into any of the above categories were counted (grey fills in Figure 5A), but not further analyzed. Of the categorized genes, ∼2/3 fell into the ‘mainly transcription’ class, while the remainders were evenly divided between the ‘mainly exosome degradation’ and ‘exosome degradation and transcription’ classes. We initially subdivided these three classes based on whether they were up- or down-regulated, but found no substantial differences in the analyses below based on this subclassification.

In the three categories, most genes were protein coding, although the class producing exosome sensitive RNAs contained a larger fraction of lncRNAs (Figure 5B), in agreement with the known involvement of the exosome in the turnover of these transcripts (34,64,65,68). For most gene types, the group of RNAs regulated by a combination of exosome decay and transcription constituted an intermediate between the ‘mainly degradation’ and ‘mainly transcription’ classes.

Our finding that a high proportion of transcripts from protein coding genes were exosome targets was surprising as these RNAs would be expected to be exported from the nucleus without engaging with the nuclear decay machinery (69,70). Hence, to address whether the observed sensitivity could be due to the stabilization of short transcripts produced by premature transcription termination (71–73), we assessed the changes in RNA levels upon exosome depletion on the exonic, rather than the full gene, level for up-regulated and exosome-sensitive protein-coding genes. This revealed that increased RNA levels upon exosome depletion were consistent across exons. (Figure 5C, transcripts exosome-sensitive in ESCs and Supplementary Figure S5C, transcripts exosome-sensitive in EBd3 cells). This implies that full-length transcripts were stabilized, which was further supported by genome browser visualizations of individual loci (Figure 5D, Supplementary Figure S5D and S5E).

To better understand why these full-length mRNAs were exosome sensitive, we compared potential features of interest between mRNAs deriving from the three established classes of genes. RNAs subjected to exosomal degradation generally exhibited both significantly lower expression levels (*P* < 1.17e–22, Mann–Whitney two-sided test) and were lower transcribed (*P* < 2.33e–5, Mann–Whitney two-sided test) (Figure 5E). Moreover, genes belonging to the ‘exosome degradation and transcription’ class showed significantly (*P* < 5.26e–56, Mann–Whitney two-sided test) higher fold changes of their RNAs between the two differentiation stages (Figure 5F), indicating that this combination of transcriptional and post-transcriptional regulatory processes yielded a more distinct response. Finally, RNAs regulated by exosomal degradation were generally shorter and contained fewer exons than RNAs whose levels were solely regulated by transcription (Figure 5G), demonstrating that exosome-regulated RNAs share characteristics with lncRNAs (74–76).

## DISCUSSION

It is well accepted that transcriptional changes contribute to gene expression regulation during cellular differentiation. Several mechanisms that lead to changes in RNA stability have also been shown to function in pluripotency control or regulation of differentiation, but the role of nuclear RNA decay in these processes has not been comprehensively investigated. Here, we reveal a substantial contribution of RNA decay by the nuclear form of the 3′-5′ ribonucleolytic RNA exosome to the alteration of RNA levels during ESC differentiation into EBs. However, although exosome-mediated RNA turnover explains several changes that cannot be explained by transcriptional regulation, there are also genes with non-correlated RNA and transcription changes, whose products do not exhibit exosome sensitivity. Thus, other RNA processing mechanisms are likely to shape the observed RNA output. Indeed, it appears that the exosome is preferentially involved in degrading lowly expressed RNAs and that this property help regulate transcriptome changes during differentiation so that RNA levels of certain genes are efficiently depleted.

A major observation from our study is that exosome and RBM7 targets tend to result from lowly expressed genes. This is in agreement with the reported low expression of NEXT targets (39). A similar trend for exosome substrates was reported in *Drosophila melanogaster* cells (77) but the mechanism underlying this phenomenon is not understood. One possibility is that a basal amount of exosome activity is ‘associated’ to each gene, which can only be overcome by a certain threshold level of expression. However, in *D. melanogaster* the exosome is recruited to elongating RNA polymerase II complexes and active regions of chromatin (77,78), which would seemingly recruit more exosome to highly transcribed genes. Another possibility is that the nuclear exosome is a limiting factor and therefore lowly expressed RNAs might encounter a higher effective concentration per molecule. However, at odds with this suggestion, highly expressed RNAs can also be very efficiently targeted by the exosome (e.g. (39)).

According to data presented here, the RNA exosome often contributes to depleting RNA levels from genes that have become transcriptionally repressed. Such depletion is not solely a function of low expression, but seems to be an additional regulatory component on top of the general correlation between low transcription levels and high exosome sensitivity. This is reminiscent of how nuclear decay participates in the rapid remodeling of gene expression in *Saccharomyces cerevisiae* upon diauxic shock. During such glucose starvation, genes that are repressed exhibit a decrease in RNA polymerase occupancy while their RNAs concomitantly are bound by exosome co-factors (79). While no specific features were found to account for such preferential exosome targeting in *S. cerevisiae*, the exosome targets identified in this study are overrepresented by mRNAs that are shorter and contain fewer exons than their stable counterparts. These are characteristics usually attributed to lncRNAs (74–76), which are well known exosome targets (34,64,65,71,80). We therefore speculate that these features, at least partially, explain transcript decay. In fact, annotation-independent classification of RNAs based on their biochemical or metabolic features resulted in groups containing both mRNAs and lncRNAs, demonstrating their shared metabolism (16,67). As mRNAs, and many lncRNAs, are polyadenylated, this feature could direct them for decay through the binding of PABPN1, which connects transcripts to the RNA exosome (39,81–83). Presumably, nuclear-retained transcripts are subject to such decay due to their longer exposure to nuclear RNA degradation enzymes (84,85). In agreement with this, RNAs exhibiting high decay rates also tended to be more nuclear (16). RNA nuclear retention could also be a consequence of its low splicing efficiency, which was indeed a feature for some RNAs with short half-lives (16).

Although features distinguishing RNAs for degradation likely include their processing efficiency, length and number of exons, these alone cannot explain the exosome specificity for lowly expressed RNAs or for RNAs that are lower expressed in one of the cell states. Therefore, other elements/factors must be involved in the specific targeting when gene expression is downregulated. Understanding these mechanisms remains an important research line for the future.

## DATA AVAILABILITY

Data available at GEO Series accession number: GSE115727

CAGE SubSeries: GSE115710

PROseq SubSeries: GSE115713

RNAseq SubSeries: GSE115714

## Supplementary Material

Supplementary Data
