# Supplementary material for: The RNA exosome contributes to gene expression regulation during stem cell differentiation
Source: Nucleic Acids Res. 2018 Sep 13;46(21):11502–13. doi: 10.1093/nar/gky817 (PMC6265456; doi:10.1093/nar/gky817)
Supplement: Supplementary Data [file gky817_supplemental_files.pdf]

Supplementary figure legends

Figure S1.

(A) Light microscopy images of EBd3 cells derived from ESCs treated with the indicated shRNAs. The scale bar on the lower right corner of the images represents 200 µm. (B) Levels of indicated transcripts relative to *Actb* mRNA as measured by RT-qPCR on total RNA harvested from the indicated cell states subjected to control (dark grey) or RRP40 (light grey) shRNA infection. Three independent biological replicates are shown. Error bars display standard deviations from 3 technical PCR replicates. (C) Levels of *Exosc3* mRNA measured by RT-qPCR as in (B) but displayed as the fraction of transcript in RRP40 depleted *vs.* control samples. Results from both ESC and EBd3 states are shown for all the triplicate samples. Error bars display standard deviations from 3 technical PCR replicates. (D) Replicate Pearson correlation coefficients between RNAseq (top), CAGE (mid) and PROseq (bottom) samples from ESC (left) and EBd3 (right) states. (E) Left: Normalized RNAseq values in $\log_2$ scale of 39 selected ESC transcript markers (see Table S1) in RRP40 depleted (y-axis) *vs.* control (x-axis) ESC samples. Right: Normalized RNAseq values in $\log_2$ scale of 56 selected day 3 lineage transcript markers (see Table S1) in RRP40 depleted (y-axis) *vs.* control (x-axis) EBd3 samples.

Figure S2.

(A-B) RNAseq $\log_2$ fold changes between RRP40-depleted and control cells for RNAs from all GENCODE annotated genes. Transcripts were quintile-stratified based on their PROseq values in control ESC (A) or EBd3 (B) states as in Figure 2. (C-D) As in (A-B), but for GENCODE annotated protein coding genes only. (E-H) Equivalent to panels (A-D), but transcripts are quintile-stratified based on RNAseq values in the respective control samples. (I) Western blotting analysis of RBM7 levels in ESCs treated with the indicated shRNAs and differentiated for 0 (left) or 3 (right) days. Tubulin was used as a loading control. Each control and RBM7-depleted lane derive from the same blot, but was cut as indicated by the dashed line. (J-K) Distributions of RBM7 sensitivities calculated from RNAseq data (see Methods) for RNAs from all GENCODE annotated genes, quintile-stratified based on their normalized PROseq values in control ESC (J) or EBd3 (K) cells. (L-M) As in (J-K) but for RNAs from GENCODE-annotated protein-coding genes only. (N-Q) Equivalent to panels (A-D) but with quintile-stratification based on normalized RNAseq values in

the respective control samples. For all plots, the number of genes/RNAs (N) included is indicated above each panel. Quintile ranges are indicated to the right of each plot.

Figure S3.

(A-B) As in Fig. 3A and 3B, left panels, but using normalized CAGE values for TSSs from control EBd3 (y-axis) and ESC (x-axis) states and coloring exosome sensitivity based on CAGE data from ESC (A) or EBd3 (B) samples. (C-D) $\log_{10}$ normalized RNAseq values for RNAs from all GENCODE-annotated genes in control EBd3 (y-axis) and control ESC (x-axis) states, coloured according to their RBM7 sensitivity in the ESC state (C) or the EBd3 state (D) as calculated from RNAseq data (as shown on the legend to the right: purple denotes the most RBM7-sensitive RNAs). (E-F) As in C-D, but using normalized CAGE values for TSSs from control EBd3 (y-axis) and ESC (x-axis) states and coloring RBM7 sensitivity based on CAGE data from ESC (E) or EBd3 (F). (G-H) As in C-D, but signals on x- and y-axes are based on PROseq data. For all plots, the number of genes/RNAs (N) included is indicated above each panel.

Figure S4.

(A) Density plots of RNAseq-calculated exosome sensitivities from ESC (blue line) and EBd3 (red line), analyzing transcripts downregulated in EBd3 with correlation between RNAseq and PROseq signals (bottom-left quadrant from Fig. 4B). (B) Density plots as in (A) but analyzing transcripts downregulated in EBd3 that do not show correlation between RNAseq and PROseq signals (bottom-right quadrant from Fig. 4B). The number of transcripts analyzed (N) is indicated above each panel. Note that the upper illustration in the figure is taken from Fig. 4B.

Figure S5.

(A) RNAseq and PROseq $\log_2$ normalized values for specific RNAs/genes (as indicated above each plot) from the ESC and EBd3 samples as indicated. Data presented are averages from all replicates. Above each panel, the behaviour and suggested type of regulation of each group of genes is indicated. (B) Fold changes, as measured by RT-qPCR, of indicated exosome mRNA targets in ESCs (left panel) and EBd3 cells (right panel). RNA levels were normalized to *Actb* mRNA levels and plotted as the ratio between RRP40 depleted and control cells. The average from three biological replicates is depicted. Error bars represent the standard deviation

from three biological replicates. Note that RNA stabilization in the EBd3 state is generally lower, in line with the lower efficiency of the RRP40 depletion in these cells. (C) RNAseq $\log_2$ fold changes between RRP40 depleted and control EBd3 samples along each exon (white) and intron (grey) of pre-mRNAs from the classes ‘mainly exosome degradation’ and ‘combination transcription and exosome degradation’. The number of transcripts analyzed (N) is indicated above the panel. (D) Genome browser screenshot of *Krt19*, a gene which is lowly expressed and exosome sensitive in the ESC state, arranged as in Fig. 5D. For RNAseq and PROseq tracks, two different scales are indicated; the top one for the EBd3 samples and the bottom one for the ESC samples. The CAGE tracks show pooled data from the two replicates and the RNAseq and PROseq tracks show one of the replicates. (E) As in (D) but visualizing *Cxcr4*, a gene which is lowly expressed and exosome sensitive in the EBd3 state. For CAGE and RNAseq tracks, two different scales are indicated for EBd3 (top) and ESC (bottom) samples.

Supplementary tables

Table S1. Markers used in Fig. S1E

Table S2. Primers used for RT-qPCR analyses

Table S3. List of spike-ins used in the RNAseq experiments with its corresponding sequence

Figure S1

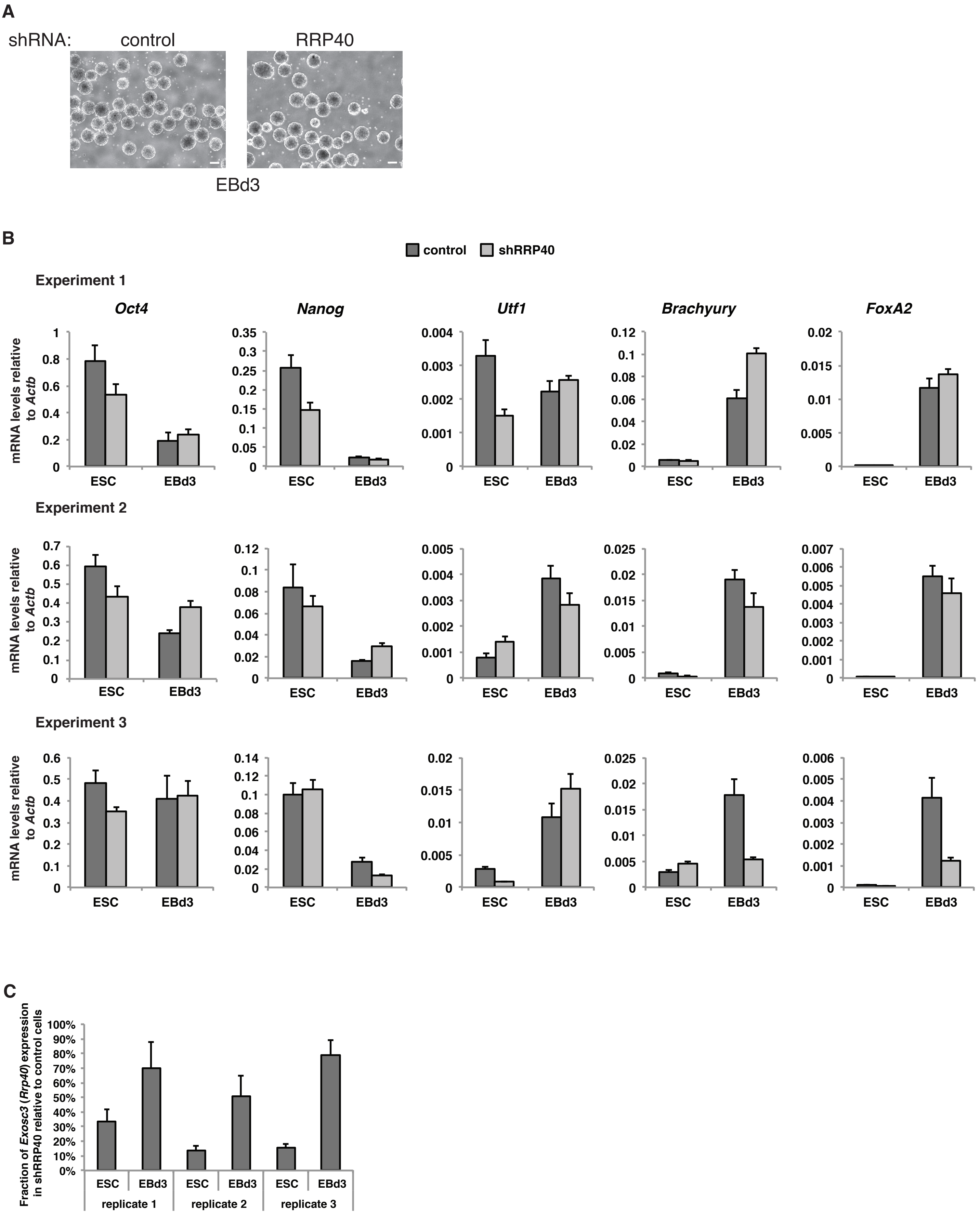

A
shRNA:
control
RRP40
EBd3
B
control
shRRP40
Experiment 1
Oct4
Nanog
Utf1
Brachyury
FoxA2
mRNA levels relative to Actb
ESC
EBd3
Experiment 2
Experiment 3
C
Fraction of Exosc3 (Rrp40) expression in shRRP40 relative to control cells
replicate 1
replicate 2
replicate 3

Figure S1

D

RNAseq ESC

	RRP40 R1	RRP40 R2	RRP40 R3	control R1	control R2	control R3
RRP40 R1	1	0.998	0.998	0.995	0.992	0.992
RRP40 R2		1	1	0.995	0.996	0.995
RRP40 R3			1	0.995	0.995	0.995
control R1				1	0.997	0.997
control R2					1	1
control R3						1

RNAseq EBd3

	RRP40 R1	RRP40 R2	RRP40 R3	control R1	control R2	control R3
RRP40 R1	1	0.998	0.999	0.997	0.997	0.996
RRP40 R2		1	0.997	0.995	0.994	0.993
RRP40 R3			1	0.996	0.996	0.997
control R1				1	0.999	0.999
control R2					1	0.998
control R3						1

CAGE ESC

	RRP40 R1	RRP40 R2	control R1	control R2
RRP40 R1	1	0.97	0.947	0.928
RRP40 R2		1	0.963	0.932
control R1			1	0.942
control R2				1

CAGE EBd3

	RRP40 R1	RRP40 R2	control R1	control R2
RRP40 R1	1	0.983	0.977	0.976
RRP40 R2		1	0.975	0.966
control R1			1	0.977
control R2				1

PROseq ESC

	RRP40 R1	RRP40 R2	control R1	control R2
RRP40 R1	1	0.981	0.943	0.969
RRP40 R2		1	0.935	0.969
control R1			1	0.98
control R2				1

PROseq EBd3

	RRP40 R1	RRP40 R2	control R1	control R2
RRP40 R1	1	0.989	0.969	0.991
RRP40 R2		1	0.951	0.971
control R1			1	0.966
control R2				1

E

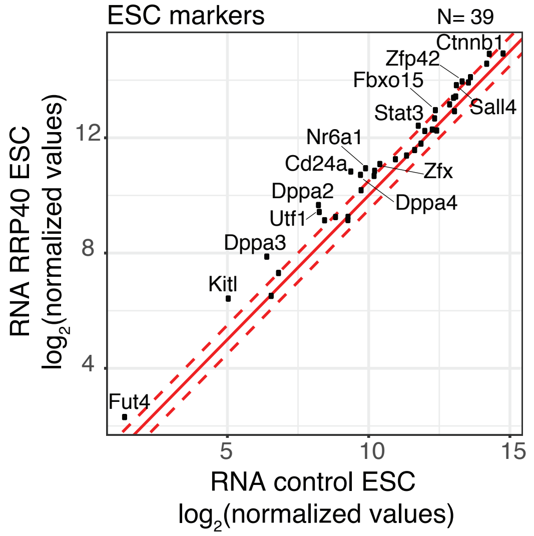

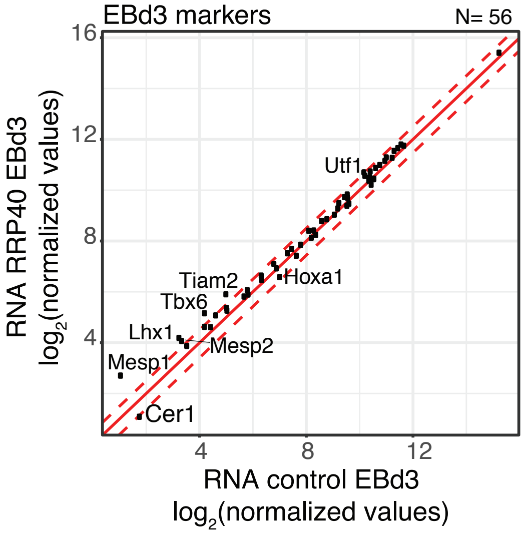

Figure S2

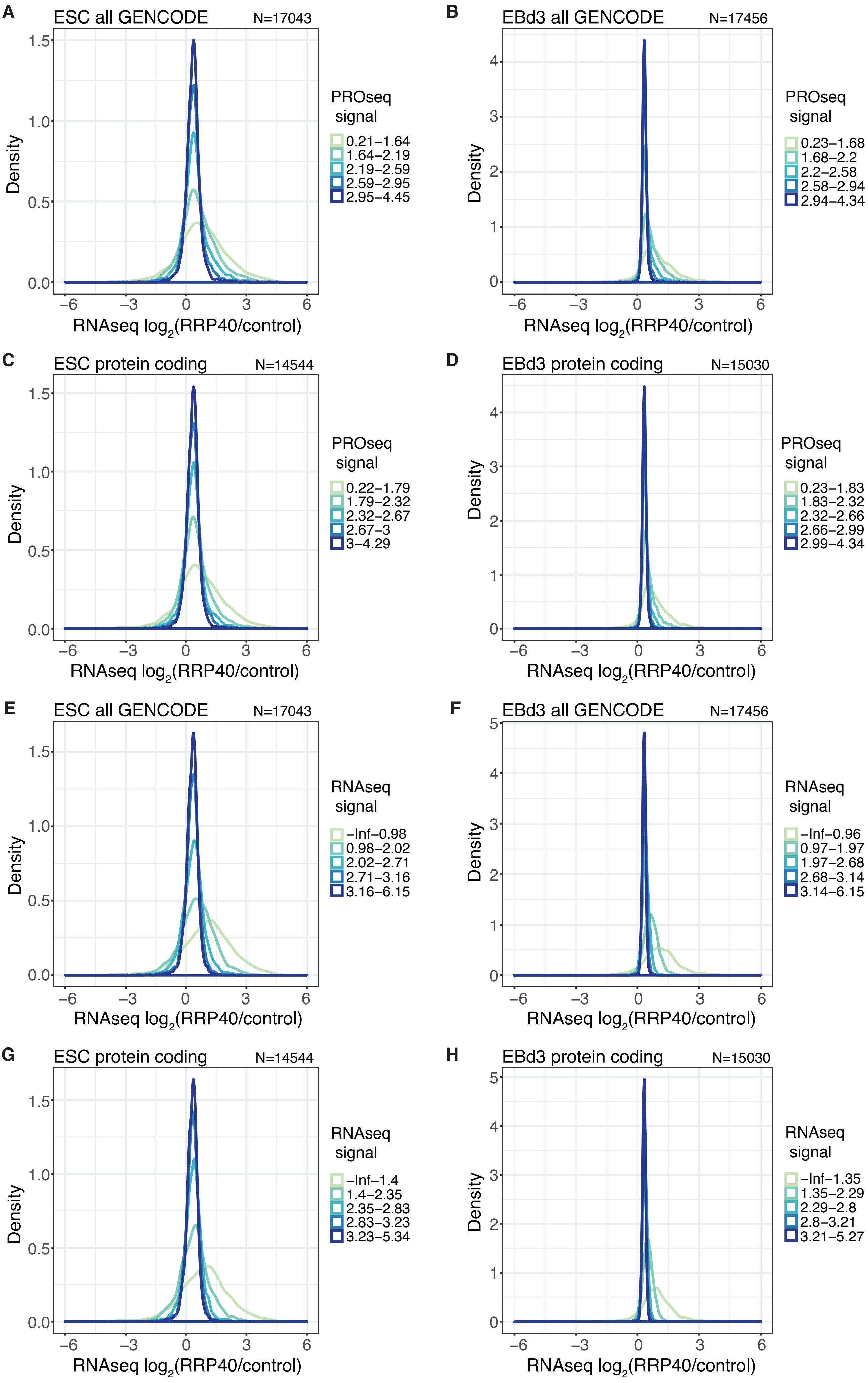
A
ESC all GENCODE
N=17043
Density
RNAseq log2(RRP40/control)
PROseq signal
0.21−1.64
1.64−2.19
2.19−2.59
2.59−2.95
2.95−4.45
B
EBd3 all GENCODE
N=17456
Density
RNAseq log2(RRP40/control)
PROseq signal
0.23−1.68
1.68−2.2
2.2−2.58
2.58−2.94
2.94−4.34
C
ESC protein coding
N=14544
Density
RNAseq log2(RRP40/control)
PROseq signal
0.22−1.79
1.79−2.32
2.32−2.67
2.67−3
3−4.29
D
EBd3 protein coding
N=15030
Density
RNAseq log2(RRP40/control)
PROseq signal
0.23−1.83
1.83−2.32
2.32−2.66
2.66−2.99
2.99−4.34
E
ESC all GENCODE
N=17043
Density
RNAseq log2(RRP40/control)
RNAseq signal
−Inf−0.98
0.98−2.02
2.02−2.71
2.71−3.16
3.16−6.15
F
EBd3 all GENCODE
N=17456
Density
RNAseq log2(RRP40/control)
RNAseq signal
−Inf−0.96
0.97−1.97
1.97−2.68
2.68−3.14
3.14−6.15
G
ESC protein coding
N=14544
Density
RNAseq log2(RRP40/control)
RNAseq signal
−Inf−1.4
1.4−2.35
2.35−2.83
2.83−3.23
3.23−5.34
H
EBd3 protein coding
N=15030
Density
RNAseq log2(RRP40/control)
RNAseq signal
−Inf−1.35
1.35−2.29
2.29−2.8
2.8−3.21
3.21−5.27

Figure S2

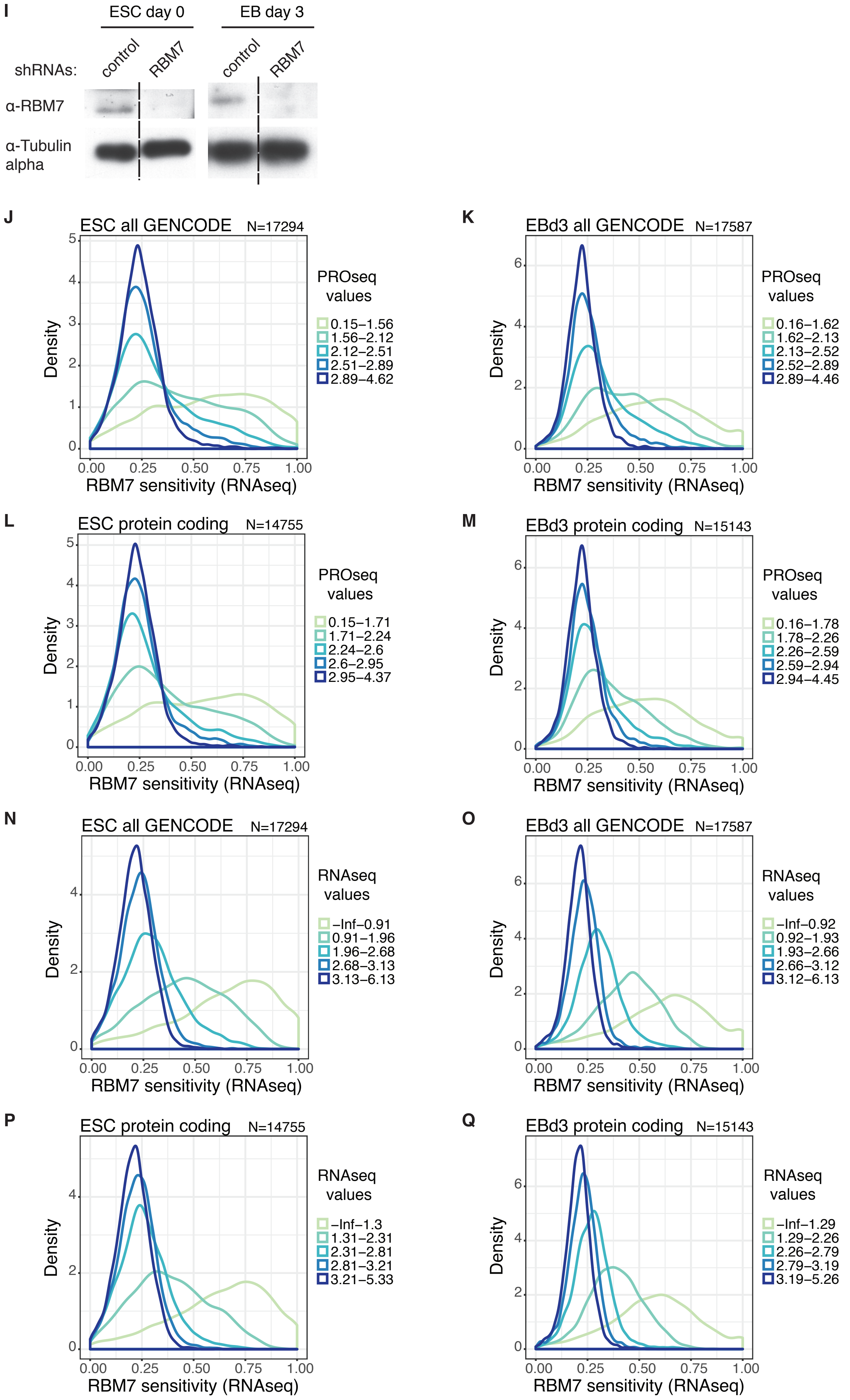
I
ESC day 0
EB day 3
shRNAs:
control
RBM7
control
RBM7
α-RBM7
α-Tubulin alpha
J
ESC all GENCODE N=17294
PROseq values
0.15−1.56
1.56−2.12
2.12−2.51
2.51−2.89
2.89−4.62
K
EBd3 all GENCODE N=17587
PROseq values
0.16−1.62
1.62−2.13
2.13−2.52
2.52−2.89
2.89−4.46
L
ESC protein coding N=14755
PROseq values
0.15−1.71
1.71−2.24
2.24−2.6
2.6−2.95
2.95−4.37
M
EBd3 protein coding N=15143
PROseq values
0.16−1.78
1.78−2.26
2.26−2.59
2.59−2.94
2.94−4.45
N
ESC all GENCODE N=17294
RNAseq values
−Inf−0.91
0.91−1.96
1.96−2.68
2.68−3.13
3.13−6.13
O
EBd3 all GENCODE N=17587
RNAseq values
−Inf−0.92
0.92−1.93
1.93−2.66
2.66−3.12
3.12−6.13
P
ESC protein coding N=14755
RNAseq values
−Inf−1.3
1.31−2.31
2.31−2.81
2.81−3.21
3.21−5.33
Q
EBd3 protein coding N=15143
RNAseq values
−Inf−1.29
1.29−2.26
2.26−2.79
2.79−3.19
3.19−5.26
Density
RBM7 sensitivity (RNAseq)
0.00
0.25
0.50
0.75
1.00

Figure S3

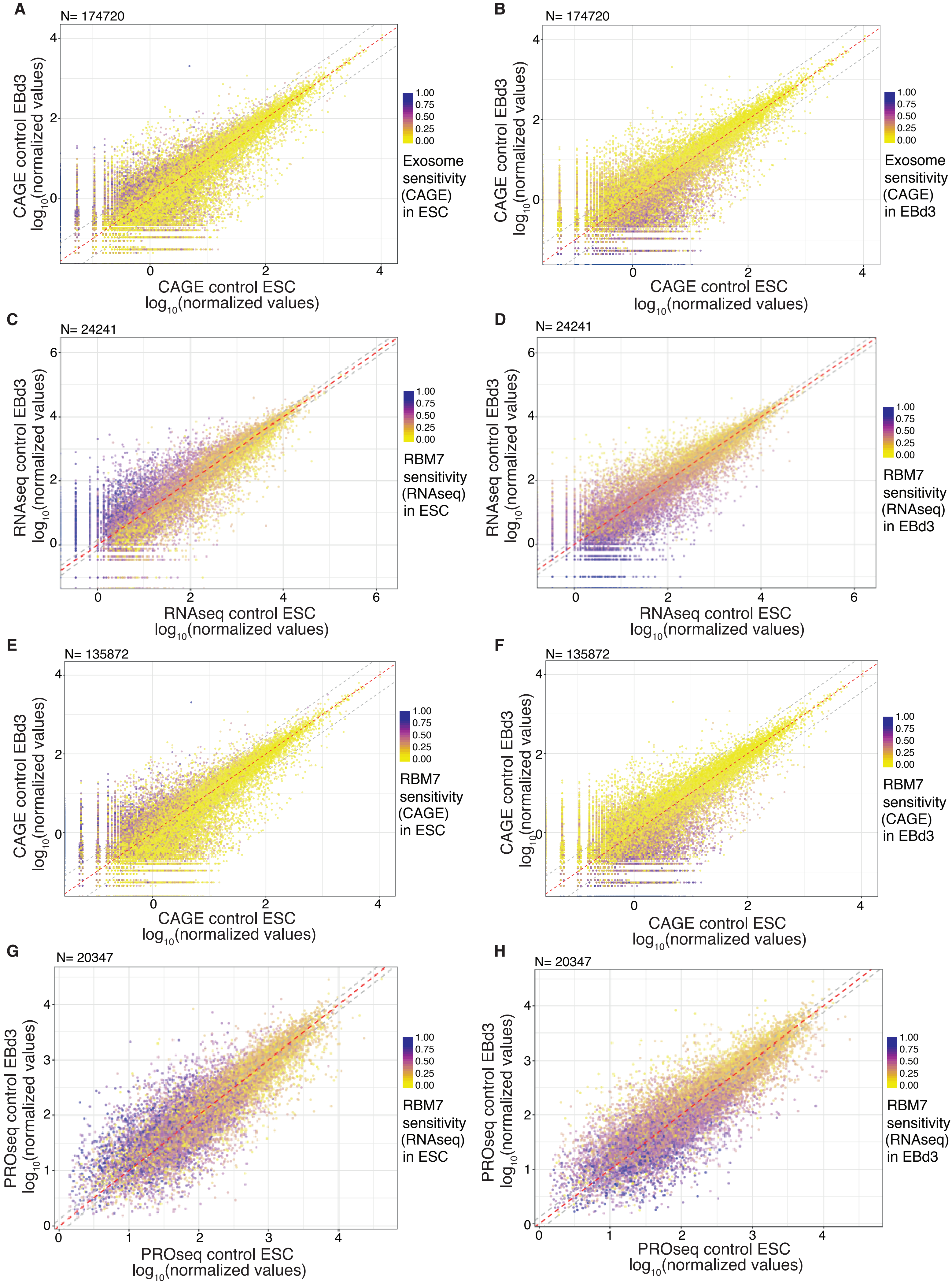
A
N= 174720
CAGE control EBd3 log10(normalized values)
CAGE control ESC log10(normalized values)
Exosome sensitivity (CAGE) in ESC
B
N= 174720
CAGE control EBd3 log10(normalized values)
CAGE control ESC log10(normalized values)
Exosome sensitivity (CAGE) in EBd3
C
N= 24241
RNAseq control EBd3 log10(normalized values)
RNAseq control ESC log10(normalized values)
RBM7 sensitivity (RNAseq) in ESC
D
N= 24241
RNAseq control EBd3 log10(normalized values)
RNAseq control ESC log10(normalized values)
RBM7 sensitivity (RNAseq) in EBd3
E
N= 135872
CAGE control EBd3 log10(normalized values)
CAGE control ESC log10(normalized values)
RBM7 sensitivity (CAGE) in ESC
F
N= 135872
CAGE control EBd3 log10(normalized values)
CAGE control ESC log10(normalized values)
RBM7 sensitivity (CAGE) in EBd3
G
N= 20347
PROseq control EBd3 log10(normalized values)
PROseq control ESC log10(normalized values)
RBM7 sensitivity (RNAseq) in ESC
H
N= 20347
PROseq control EBd3 log10(normalized values)
PROseq control ESC log10(normalized values)
RBM7 sensitivity (RNAseq) in EBd3
1.00
0.75
0.50
0.25
0.00

Figure S4

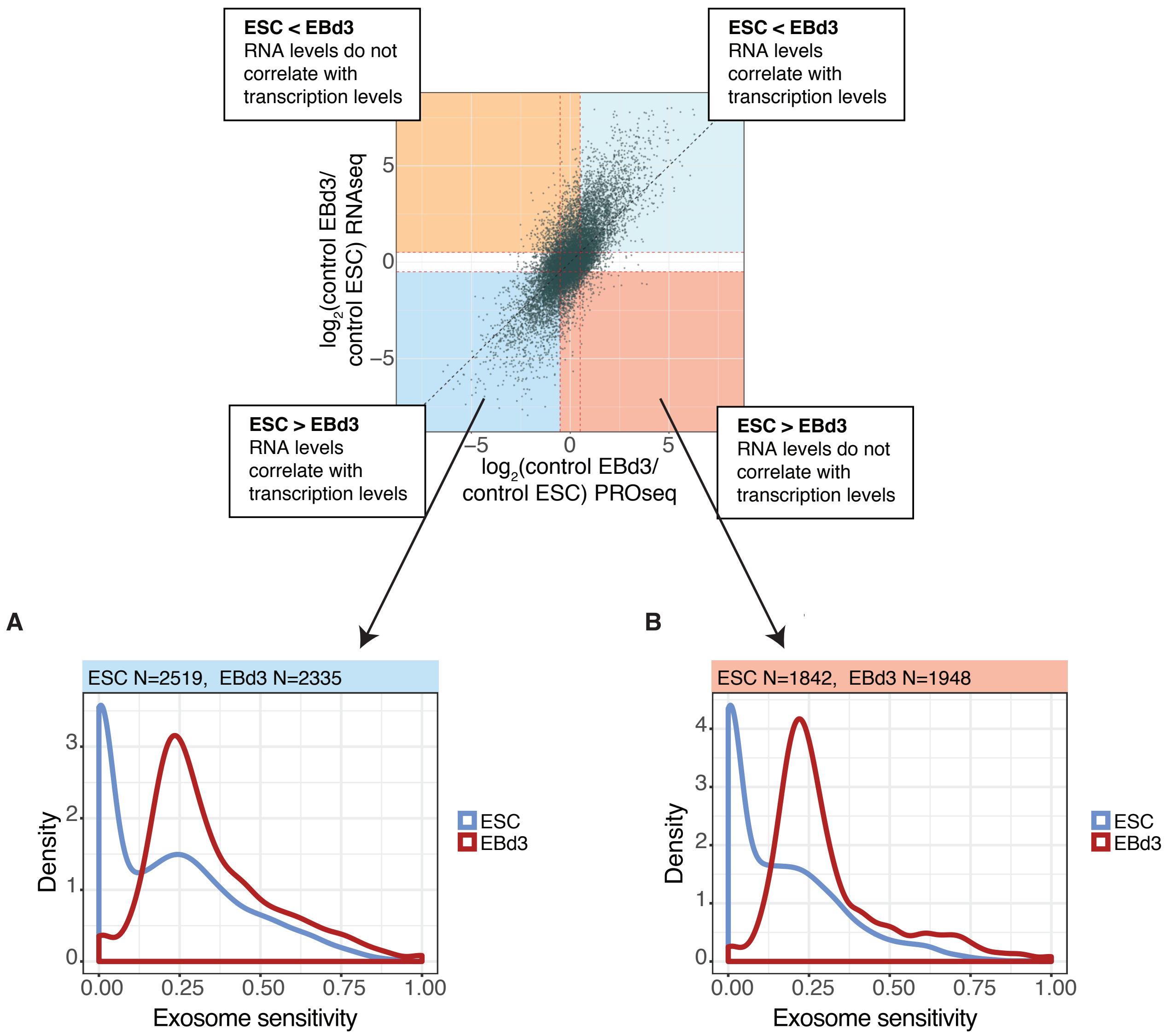
ESC < EBd3
RNA levels do not correlate with transcription levels
ESC < EBd3
RNA levels correlate with transcription levels
ESC > EBd3
RNA levels correlate with transcription levels
ESC > EBd3
RNA levels do not correlate with transcription levels
log2(control EBd3/ control ESC) RNAseq
log2(control EBd3/ control ESC) PROseq
−5
0
5
A
B
ESC N=2519, EBd3 N=2335
ESC N=1842, EBd3 N=1948
Density
Exosome sensitivity
0.00
0.25
0.50
0.75
1.00
ESC
EBd3

Figure S5

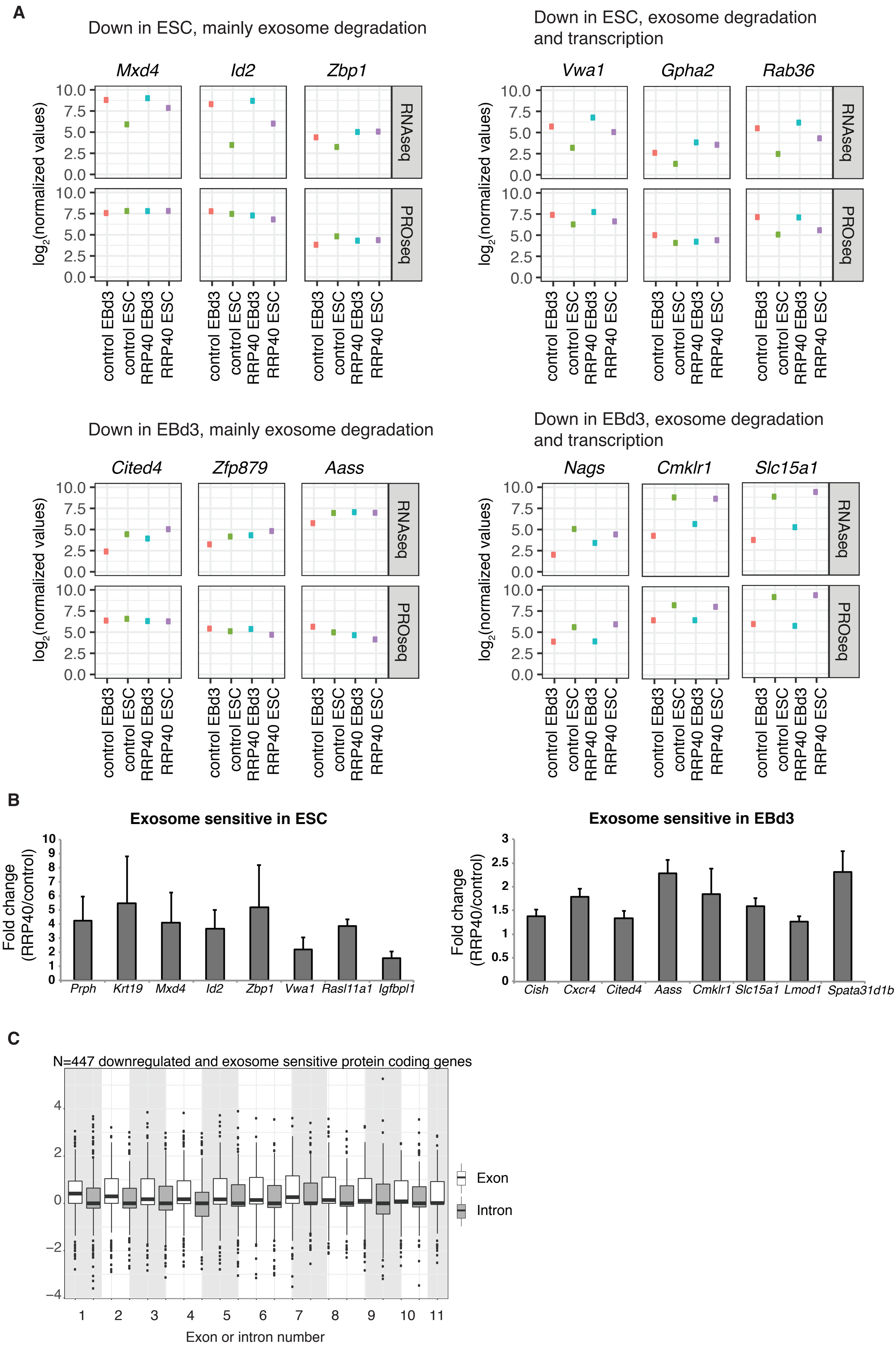
A
Down in ESC, mainly exosome degradation
Mxd4
Id2
Zbp1
Down in ESC, exosome degradation and transcription
Vwa1
Gpha2
Rab36
RNAseq
PROseq
log2(normalized values)
control EBd3
control ESC
RRP40 EBd3
RRP40 ESC
Down in EBd3, mainly exosome degradation
Cited4
Zfp879
Aass
Down in EBd3, exosome degradation and transcription
Nags
Cmklr1
Slc15a1
B
Exosome sensitive in ESC
Fold change (RRP40/control)
Prph
Krt19
Mxd4
Id2
Zbp1
Vwa1
Rasl11a1
Igfbpl1
Exosome sensitive in EBd3
Cish
Cxcr4
Cited4
Aass
Cmklr1
Slc15a1
Lmod1
Spata31d1b
C
N=447 downregulated and exosome sensitive protein coding genes
Exon
Intron
Exon or intron number

Figure S5

D Down in ESC, exosome degradation and transcription

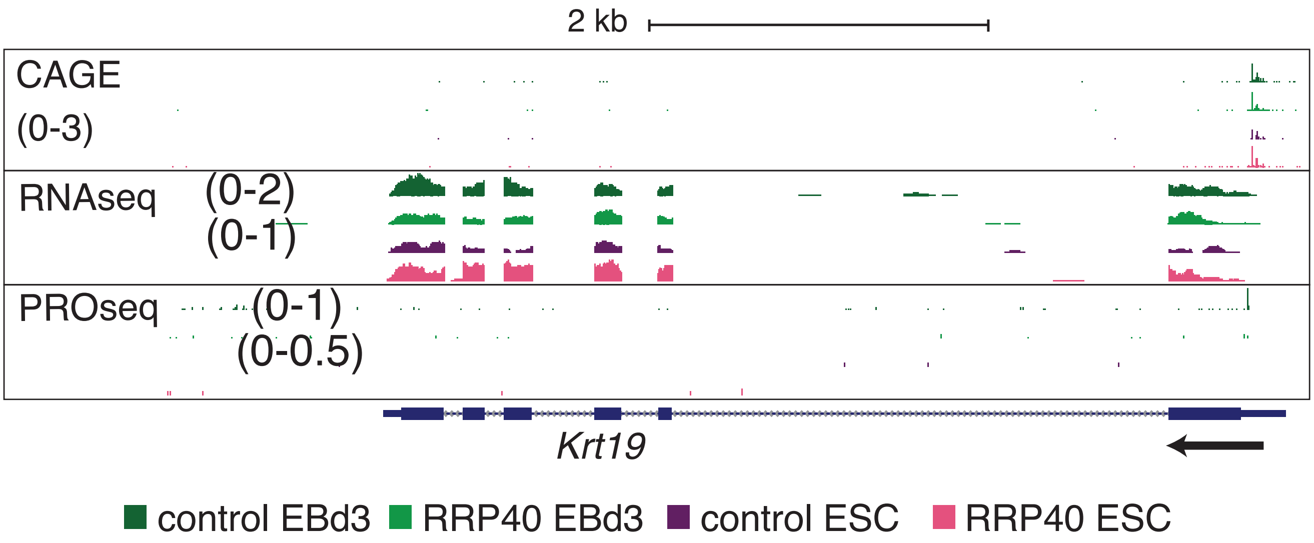

E Down in EBd3, exosome degradation and transcription

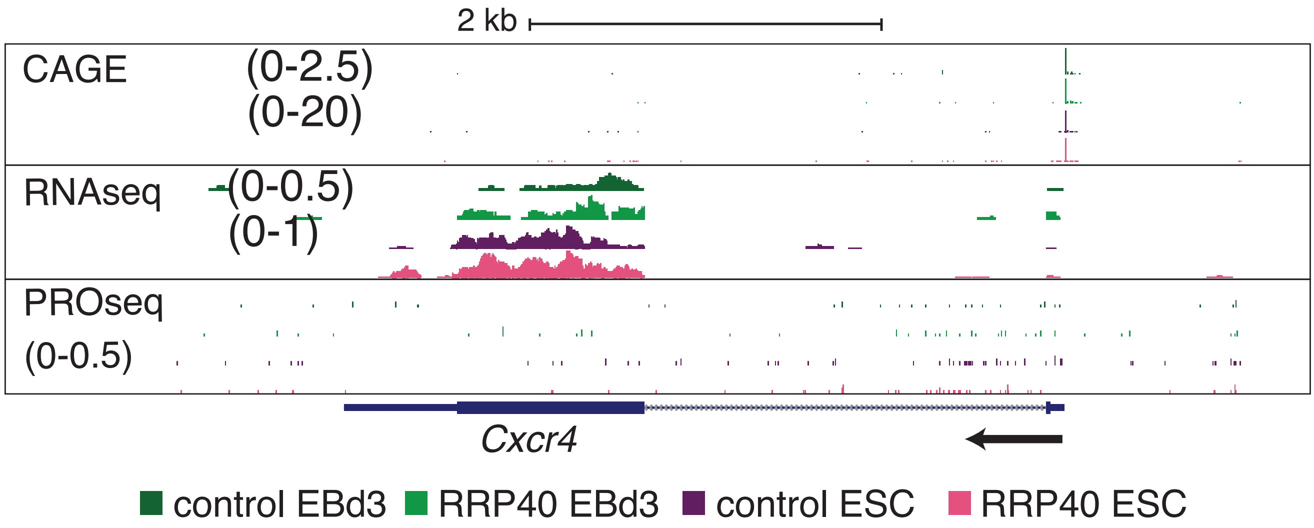

Table S1. Markers used in Fig. S1E

**ESC markers**		**EB day 3 markers**	
**Gene ID**	**Gene name**	**Gene ID**	**Gene name**
ENSMUSG00000000303.12	*Cdh1*	ENSMUSG00000000125.5	*Wnt3*
ENSMUSG00000001910.4	*Nacc1*	ENSMUSG00000000142.15	*Axin2*
ENSMUSG00000003032.8	*Klf4*	ENSMUSG00000001211.14	*Agpat3*
ENSMUSG00000004040.16	*Stat3*	ENSMUSG00000005225.15	*Plekha8*
ENSMUSG00000005672.12	*Kit*	ENSMUSG00000005503.8	*Evx1*
ENSMUSG00000006932.16	*Ctnnb1*	ENSMUSG00000012282.2	*Wnt8a*
ENSMUSG00000012396.12	*Nanog*	ENSMUSG00000018698.15	*Lhx1*
ENSMUSG00000019966.17	*Kitl*	ENSMUSG00000018924.6	*Alox15*
ENSMUSG00000020167.14	*Tcf3*	ENSMUSG00000020577.15	*Tspan13*
ENSMUSG00000020717.19	*Pecam1*	ENSMUSG00000021068.15	*Nin*
ENSMUSG00000021540.16	*Smad5*	ENSMUSG00000021095.5	*Gsc*
ENSMUSG00000023980.6	*Taf8*	ENSMUSG00000021765.7	*Fst*
ENSMUSG00000024406.16	*Pou5f1*	ENSMUSG00000022528.7	*Hes1*
ENSMUSG00000024515.13	*Smad4*	ENSMUSG00000023800.14	*Tiam2*
ENSMUSG00000025809.15	*Itgb1*	ENSMUSG00000024308.14	*Tapbp*
ENSMUSG00000027111.15	*Itga6*	ENSMUSG00000024565.8	*Sall3*
ENSMUSG00000027547.17	*Sall4*	ENSMUSG00000024987.5	*Cyp26a1*
ENSMUSG00000029086.15	*Prom1*	ENSMUSG00000025219.13	*Fgf8*
ENSMUSG00000030342.8	*Cd9*	ENSMUSG00000026497.7	*Mixl1*
ENSMUSG00000031681.14	*Smad1*	ENSMUSG00000027478.15	*Dnmt3b*
ENSMUSG00000032011.4	*Thy1*	ENSMUSG00000027797.15	*Dclk1*
ENSMUSG00000032494.12	*Tdgf1*	ENSMUSG00000028039.11	*Efna3*
ENSMUSG00000032679.12	*Cd59a*	ENSMUSG00000028532.14	*Cachd1*
ENSMUSG00000034391.10	*Fbxo15*	ENSMUSG00000029337.2	*Fgf5*
ENSMUSG00000045005.9	*Fzd5*	ENSMUSG00000029646.3	*Cdx2*
ENSMUSG00000045394.8	*Epcam*	ENSMUSG00000029844.9	*Hoxa1*
ENSMUSG00000046323.8	*Dppa3*	ENSMUSG00000030543.10	*Mesp2*
ENSMUSG00000047139.8	*Cd24a*	ENSMUSG00000030544.5	*Mesp1*
ENSMUSG00000047751.9	*Utf1*	ENSMUSG00000030699.16	*Tbx6*
ENSMUSG00000049307.5	*Fut4*	ENSMUSG00000031012.17	*Cask*
ENSMUSG00000051176.5	*Zfp42*	ENSMUSG00000031342.17	*Gpm6b*
ENSMUSG00000056758.14	*Hmga2*	ENSMUSG00000031681.14	*Smad1*
ENSMUSG00000058550.14	*Dppa4*	ENSMUSG00000032446.14	*Eomes*
ENSMUSG00000060461.5	*Dppa5a*	ENSMUSG00000035021.13	*Baz1a*
ENSMUSG00000063972.13	*Nr6a1*	ENSMUSG00000036111.8	*Lmo1*
ENSMUSG00000067261.4	*Foxd3*	ENSMUSG00000036306.11	*Lzts1*
ENSMUSG00000072419.4	*Dppa2*	ENSMUSG00000036885.14	*Arhgef26*
ENSMUSG00000074637.7	*Sox2*	ENSMUSG00000036913.14	*Trim67*
ENSMUSG00000079509.10	*Zfx*	ENSMUSG00000037016.11	*Frem2*
		ENSMUSG00000037025.11	*Foxa2*
		ENSMUSG00000037347.7	*Chst7*
		ENSMUSG00000038192.5	*Cer1*
		ENSMUSG00000039316.14	*Rftn1*
		ENSMUSG00000042821.7	*Snai1*
		ENSMUSG00000045658.16	*Pid1*
		ENSMUSG00000046402.10	*Rbp1*
		ENSMUSG00000047751.9	*Utf1*
		ENSMUSG00000048562.6	*Sp8*
		ENSMUSG00000050105.5	*Grrp1*
		ENSMUSG00000051851.5	*Cxx1c*
		ENSMUSG00000062327.10	*T*
		ENSMUSG00000062393.13	*Dgkk*
		ENSMUSG00000070880.10	*Gad1*
		ENSMUSG00000075304.2	*Sp5*
		ENSMUSG00000079442.12	*St6galnac4*
		ENSMUSG00000086126.1	*Evx1os*

Table S2. Primers used for RT-qPCR analyses

Gene	Sequence (5' to 3')
Oct4	GCTCACCCTGGGCGTTCTC<br>GGCCGCAGCTTACACATGTTC
Nanog	CCTCCAGCAGATGCAAGAACTC<br>CTTCAACCACTGGTTTTTCTGCC
Utf1	GTCCCTCTCCGCGTTAGCAC<br>GTGGAAGAACTGAATCTGAGCGC
Brachyury	GAACGGCAGGAGGATGTTCCC<br>AGCGGTGGTTGTCAGCCGTC
FoxA2	GATGGAAGGGCACGAGCC<br>GTATGTGTTCATGCCATTCATCCC
Rrp40	GATGTTGGAGGGAGTGAGCC<br>CTCCAACCTGCACATTTGGTC
Actb	TCCACACCCGCCACCAGTTCG<br>CACATGCCGGAGCCGTTGTC
Prph	ACAATTGAGACCCGGGATGG<br>CTTGTCCAGGTCACTGTGCT
Krt19	TCAGTACGCATTGGGTCAGG<br>GAGGACGAGGTCACGAAGC
Mxd4	GAGCCTTCTGAAGCGTGCTA<br>TTCCTTGATGCTCAGTGCCC
Id2	GAAAGCCTTCAGTCCGGTGA<br>AGACTCATCGGGTCGTCCA
Zbp1	ATGACGGACAGACGTGGAAG<br>AATCGCAGGGGACTCTTGTG
Vwa1	TCGTCAAAACTCTCCCCAGC<br>GCTTCCCCTACTCCCCAAAC
Rasl11a1	CTCGTCTATGTGGAGGGGGA<br>TCTGCCCAGTGCACACTTTT
Igfbpl1	AGCTGACTCGTGACTTGGTG<br>CCCTCCCTTCATAACACGCA
Cish	TGTCGGCTAGTCATCAACCG<br>GGGGTACTGTCGGAGGTAGT
Cxcr4	TACCTCGCTATTGTCCACGC<br>CCAGACGCCCACATAGACTG
Cited4	TGACAGTTGGGTCTCGCTTC<br>TCCGAAGGCTGGTTCAGTTC
Aass	GAGAAGCATACCCTGCCCTC<br>GGAGGAGCGGGAAATTCCAA
Cmklr1	GCCAACATACACGATGTCGC<br>TTGCGGTAACTTCCTCACCC
Slc15a1	TCCCACCCAACACTGTGAAC<br>GACAGAGAAGACCACCTCGC
Lmod1	ACCCTCCACCTGATATGCCT<br>GCATCAACCCTTCAGGTCCA
Spata31d1b	TGTGCTTGCTTCTCCACCAA<br>TAATGGAAACAAACCGCGCC

Table S3. List of spike-ins used in the RNAseq experiments with its corresponding sequence

**T7_CAT**
GGGCGAATTGAATTTAGCGGCCGCGAATTCGCCCTTTCGACCAGCTTGGC<br>GAGATTTTCAGGAGCTAAGGAAGCTAAAATGGAGAAAAAAATCACTGGAT<br>ATACCACCGTTGATATATCCCAATGGCATCGTAAAGAACATTTTGAGGCA<br>TTTCAGTCAGTTGCTCAATGTACCTATAACCAGACCGTTCAGCTGGATAT<br>TACGGCCTTTTTAAAGACCGTAAAGAAAAATAAGCACAAGTTTTATCCGG<br>CCTTTATTCACATTCTTGCCCGCCTGATGAATGCTCATCCGG
**T3_Neo_pA**
GGGAGATCTCGTGATGGCAGGTTGGGCGTCGCTTGGTCGGTCATTTCGAA<br>CCCCAGAGTCCCGCTCAGAAGAACTCGTCAAGAAGGCGATAGAAGGCGAT<br>GCGCTGCGAATCGGGAGCGGCGATACCGTAAAGCACGAGGAAGCGGTCAG<br>CCCATTCGCCGCCAAGCTCTTCAGCAATATCACGGGTAGCCAACGCTATG<br>TCCTGATAGCGGTCCGCCACACCCAGCCGGCCACAGTCGATGAATCCAGA<br>AAAAAAAAAAAAAAAAAAAAAAAAAAGGGCGAATTCGCGGCC
**T3_CAT**
GGGACTAGTCCTGCAGGTTTAAACGAATTCGCCCTTCATGGTGAAAACGG<br>GGGCGAAGAAGTTGTCCATATTGGCCACGTTTAAATCAAAACTGGTGAAA<br>CTCACCCAGGGATTGGCTGAGACGAAAAACATATTCTCAATAAACCCTTT<br>AGGGAAATAGGCCAGGTTTTCACCGTAACACGCCACATCTTGCGAATATA<br>TGTGTAGAAACTGCCGGAAATCGTCGTGGTATTCACTCCAGAGCGATGAA<br>AACGTTTCAGTTTGCTCATGGAAAACGGTGTAACAAGGGTGAACACTATC<br>CCATATCACCAGCTCACCGTCTTTCATTGCCATACGGAATTCCGG
**Sp6_EGFP**
GAATAGGGCCCTCTAGGCGGCCGCTCTAGACTCGAGTCGACCCGGGAATT<br>CGGATCCAAGCTTCTTGTACAGCTCGTCCATGCCGAGAGTGATCCCGGCG<br>GCGGTCACGAACTCCAGCAGGACCATGTGATCGCGCTTCTCGTTGGGGTC<br>TTTGCTCAGGGCGGACTGGGTGCTCAGGTAGTGGTTGTCGGGCAGCAGCA<br>CGGGGCCGTCGCCGATGGGGGTGTTCTGCTGGTAGTGGTCGGCGAGCTGC<br>ACGCTGCCGTCCTCGATGTTGTGGCGGATCTTGAAGTTCACCTTGATGCC<br>GTTCTTCTGCTTGTCGGCCATGATATAGACGTTGTGGCTGTTGTAGTTGT<br>ACTCCAGCTTGTGCCCCAGGATGTTGCCGTCCTCCTTGAAGT
**T7_EGFP_pA**
GGGAGACCCAAGCTTGTCGCCACCATGGTGAGCAAGGGCGAGGAGCTGTT<br>CACCGGGGTGGTGCCCATCCTGGTCGAGCTGGACGGCGACGTAAACGGCC<br>ACAAGTTCAGCGTGTCCGGCGAGGGCGAGGGCGATGCCACCTACGGCAAG<br>CTGACCCTGAAGTTCATCTGCACCACCGGCAAGCTGCCCGTGCCCTGGCC<br>CACCCTCGTGACCACCCTGACCTACGGCGTGCAGTGCTTCAGCCGCTACC<br>CCGACCACATGAAGCAGCACGACTTCTTCAAGTCCGCCATGCCCGAAGGC<br>TACGTCCAGGAGCGCACCATCTTCTTCAAGGACGACGGCAACTACAAGAC<br>CCGCGCCGAGGTGAAGTTCGAGGGCGACACCCTGGTGAACCGCATCGAGC<br>TGAAGGGCATCAAAAAAAAAAAAAAAAAAAAAAAAAAAGGGCGAATTCGC<br>GGCC
**T7_LTc_pA**
TGGGAGATGGAAGACGCCAAAAACATAAAGAAAGGCCCGGCGCCATTCTA<br>TCCGCTGGAAGATGGAACCGCTGGAGAGCAACTGCATAAGGCCATGAAGA<br>GATACGCCCTGGTTCCTGGAACAATTGCTTTTACAGATGCACATATCGAG<br>GTGGACATCACTTACGCTGAGTACTTCGAAATGTCCGTTCGGTTGGCAGA

AGCTATGAAACGATATGGGCTGAATACAAATCACAGAATCGTCGTATGCA
GTGAAAACTCTCTTCAATTCTTTATGCCGGTGTTGGGCGCGTTATTTATC
GGAGTTGCAGTTGCGCCCGCGAACGACATTTATAATGAACGTGAATTGCT
CAACAGTATGGGCATTTCGCAGCCTACCGTGGTGTTCGTTTCCAAAAAGG
GGTTGCAAAAAATTTTGAACGTGCAAAAAAAAAAAAAAAAAAAAAAAAAA
AGGGCGAATTCGCGGCC

T3_LTc_pA

GGGAGACACGGCGATCTTTCCGCCCTTCTTGGCCTTTATGAGGATCTCTC
TGATTTTTCTTGCGTCGAGTTTTCCGGTAAGACCTTTCGGTACTTCGTCC
ACAAACACAACTCCTCCGCGCAACTTTTTCGCGGTTGTTACTTGACTGGC
GACGTAATCCACGATCTCTTTTTCCGTCATCGTCTTTCCGTGCTCCAAAA
CAACAACGGCGGCGGGAAGTTCACCGGCGTCATCGTCGGGAAGACCTGCG
ACACCTGCGTCGAAGATGTTGGGGTGTTGGAGCAAGATGGATTCCAATTC
AGCGGGAGCCACCTGATAGCCTTTGTACTTAATCAGAGACTTCAGGCGGT
CAACGATGAAGAAGTGTTCGTCTTCGTCCCAGTAAGCTATGTCTCCAGAA
TGTAGCCATCCATCCTTGTCAATCAAGGCGTTGGTCGCTTCCGGATTGTT
TACATAACCGGACATAATCATAGGACCTCTCACACACAGTTCGCCTCTTT
GATTAACGCCCAGCGTTTTCCCGGTATCCAGATCCACAACCTTCGCTTCA
AAAAAAAAAAAAAAAAAAAAAAAAAAGGGCGAATTCGCGGCC

Sp6_Neo_pA

GAAGAGACAGGATGAGGATCGTTTCGCATGATTGAACAAGATGGATTGCA
CGCAGGTTCTCCGGCCGCTTGGGTGGAGAGGCTATTCGGCTATGACTGGG
CACAACAGACAATCGGCTGCTCTGATGCCGCCGTGTTCCGGCTGTCAGCG
CAGGGGCGCCCGGTTCTTTTTGTCAAGACCGACCTGTCCGGTGCCCTGAA
TGAACTGCAGGACGAGGCAGCGCGGCTATCGTGGCTGGCCACGACGGGCG
TTCCTTGCGCAGCTGTGCTCGACGTTGTCACTGAAGCGGGAAGGGACTGG
CTGCTATTGGGCGAAGTGCCGGGGCAGGATCTCCTGTCATCTCACCTTGC
TCCTGCCGAGAAAGTATCCATCATGGCTGATGCAATGCGGCGGCTGCATA
CGCTTGATCCGGCTACCTGCCCATTCGACCACCAAGCGAAACATCGCATC
GAGCGAGCACGTACTCGGATGGAAGCCGGTCTTGTCGATCAGGATGATCT
GGACGAAGAGCATCAGGGGCTCGCGCCAGCCGAACTGTTCGCCAGGCTCA
AGGCGCGCATGCCCGACGGCGAGGATCTCGTCGTGACCCATGGCGATGCC
TGCTTGCCGAATATCATGGTGGAAAAAAAAAAAAAAAAAAAAAAAGGGCG
AATTCGTTTAAACCTGCAGGACTA
